# Diagnostic conversion to bipolar disorder among adolescents and young adults with major depressive disorder: a nationwide longitudinal study

**DOI:** 10.1007/s00787-024-02401-1

**Published:** 2024-03-29

**Authors:** Fan-Hsuan Kung, Chia-Kuang Tsai, Chih-Ming Cheng, Shih-Jen Tsai, Tung-Ping Su, Tzeng-Ji Chen, Ya-Mei Bai, Chih-Sung Liang, Mu-Hong Chen

**Affiliations:** 1 Department of Psychiatry, Beitou Branch, Tri-Service General Hospital, National Defense Medical Center, No. 60, Xinmin Road, Beitou District, Taipei, 11243 Taiwan; 2 Department of Neurology, Tri-Service General Hospital, National Defense Medical Center, Taipei, Taiwan; 3 Department of Psychiatry, Taipei Veterans General Hospital, No. 201, Sec. 2, Shihpai Road, Beitou District, Taipei, 11217 Taiwan; 4 Department of Psychiatry, College of Medicine, National Yang Ming Chiao Tung University, Taipei, Taiwan; 5 Department of Psychiatry, General Cheng Hsin Hospital, Taipei, Taiwan; 6 Department of Family Medicine, Taipei Veterans General Hospital, Taipei, Taiwan; 7 Institute of Hospital and Health Care Administration, National Yang Ming Chiao Tung University, Taipei, Taiwan; 8 Department of Psychiatry, National Defense Medical Center, Taipei, Taiwan

**Keywords:** Depressive disorder, Bipolar disorder, Conversion, Adolescent, Young adult

## Abstract

Although several studies have examined a diagnostic conversion from major depressive disorder (MDD) to bipolar disorder (BD), only a few studies specifically focused on adolescents and young adults who are at the peak ages of BD onset. Data from participants (N = 130,793) aged 10–29 years who were diagnosed with MDD were extracted from the Taiwan National Health Insurance Research Database. We applied demographic analyses, survival analysis, Aalen Johansen curves, and Cox regression, investigating the diagnostic conversion rate and factors that were most or less predictive of conversion. Among the adolescents and young adults with MDD, the number of participant conversion subsample is 14,187 and the conversion rate was 13.80% (95% confidence interval: 13.54–14.06%) during the 11-year follow-up. The conversion rate was highest in the first year (4.50%; 4.39–4.61%) and decreased over time. The significant predictors were younger age of diagnosis with MDD (*p* < 0.001), moderate and high antidepressant resistance (*p* < 0.001), obesity (*p* < 0.001), psychiatric comorbidities (attention-deficit/hyperactivity disorder, substance use disorder, and cluster B and C personality disorder, all *p* < 0.001), a family history of mental disorders (schizophrenia and mood disorders, all *p* < 0.05), lower monthly income (*p* < 0.001), and more mental health visits to the clinic each year (*p* < 0.001). A composite of demographic characteristics, antidepressant resistance, physical and psychiatric comorbidities, and family history significantly predicted diagnostic conversion from MDD to BD (area under the curve = 0.795, *p* < 0.001). Compared to adult population, the adolescents and young adults had different factors that were most or less predictive of conversion, which warrants further investigation.

**Supplementary Information:**

The online version contains supplementary material available at 10.1007/s00787-024-02401-1.

## Significant outcomes

1. The estimated conversion rate exhibited a tendency of gradually slowing growth, accompanied by a continuous rise in the cumulative number of conversions, albeit to a lesser extent.
2. Moderate or high antidepressant resistance, obesity, psychiatric comorbidities (ADHD, SUD (especially alcohol), and cluster B and C PDs), family history of mental disorders (schizophrenia and mood disorder) were related to the diagnostic conversion from MDD to BD in adolescent and young adult populations, and obesity and personality disorder have not been considered as risk factors of BD conversion in previous studies addressing the adult population.
3. Epilepsy, cerebrovascular disease, and TBI are factors that were less predictive of conversion from MDD to BD.

## Limitations

1. The diagnostic conversion from MDD to BD in our data could be underestimated because of less than the average years taken in meta-analytic evidence.
2. We recorded antidepressant resistance without exploring medication types and use duration.
3. We were not able to assess genetic factors and gene‒environment interactions, although we adjusted for a family history of mental disorders at baseline.
4. The association between diagnostic conversion and medication type, dosage, and duration are difficult to explore because of long-term follow-up duration.
5. We cannot completely confirm that all case included in the MDD cohort did not have a history of BD because some patients might have a previous manic episode without visiting medical service.
6. It is difficult to evaluate medication adherence in the claim database.
7. Our study utilizing the NHI system research database did not encompass personal lifestyle factors, such as sleep habits, which could potentially influence the risk of bipolar disorder.
8. It is possible that obesity could be inaccurately linked to a risk factor for BD conversion, because weight gain occurs as a side effect of mood stabilizers, which might be prescribed if there is no adequate response to antidepressant treatment.

## Introduction

Bipolar disorder (BD) is a common and disabling mental disorder that can reduce quality of life, substantially impacting several domains, such as interpersonal relationships and work productivity [32, 41]. However, accurate diagnosis of BD is challenging. The majority of first episodes in BD are depressive [3], and misdiagnosis of BD as an episode of major depressive disorder (MDD) is common. According to a national survey, as many as 69% of patients with BD initially received incorrect diagnoses [17]. Because treatment strategies and prognosis for BD and MDD differ, determining factors that were most or less predictive of diagnostic conversion from MDD to BD is important for precise diagnosis and proper therapy.

Several studies have examined the diagnostic conversion from MDD to BD, but only a few surveys have focused on adolescents and young adults at the peak ages of BD onset (between 15 and 25 years old) [2]. Recently, large national studies in England [19] and Korea [24] reported annual conversion rates and a cumulative conversion rate of 2.21–7.06% and 6.46%, respectively, among young people. Both studies reported that sex and psychotic features were risk factors for conversion to BD, and a study in Korea further suggested that the use of medication, recurrent depression, and comorbid disorders could increase the risk of the conversion from MDD to BD [24].

To date, several important risk and protective factors for BD have not been investigated in adolescents and young adults. For example, BD is highly heritable [30], but the contribution of a family history of mental disorders to conversion to BD has not been examined in adolescents and young adults. Obesity is a serious public health issue, including in the adolescent population, and it has also been associated with BD [39]. However, it remains unknown whether obesity is a risk factor for BD conversion in adolescents and young adults. Although a few studies have mentioned that personality disorders (PDs) are correlated with the risk of BD [33], specific PDs (i.e., clusters A, B, and C) have not been examined individually.

Therefore, we conducted a prospective and long-term longitudinal empirical study (follow-up: 11 years), examining the rate of conversion from MDD to BD and factors that were most or less predictive of conversion. We hypothesized that these factors would be associated with conversion from MDD to BD.

## Material and methods

### Data source

The current study used a specialized dataset of the Taiwan National Health Insurance Research Database (NHIRD) that included all medical records of insured participants with any mental disorder. The NHIRD contains comprehensive information about insured participants, including demographic data (birth date, sex, residential location, and income) and clinical visits (visit dates and medical diagnoses). The NHIRD is audited and released by the National Health Research Institute for scientific and study purposes [18, 26]. In the NHIRD, every subject is assigned a unique and anonymous identifier, and thus, a subject can be followed anonymously and continuously using the unique identifier. A specialized dataset of mental disorders including all psychiatric medical records of insured individuals between 2000 and 2011 was used to identify individuals with major depressive disorder in our study. The International Classification of Diseases, Ninth Revision, Clinical Modification (ICD-9-CM) was used to diagnose diseases during the study period. The NHIRD has been used extensively in epidemiologic studies in Taiwan [7–9, 26]. VGHTPE institutional review board approved the study protocol and waived the requirement for informed consent, because this investigation used de-identified data and no human subjects contact was required (2018-07-016AC).

### Inclusion criteria for participants with MDD

**Fig. 1:**
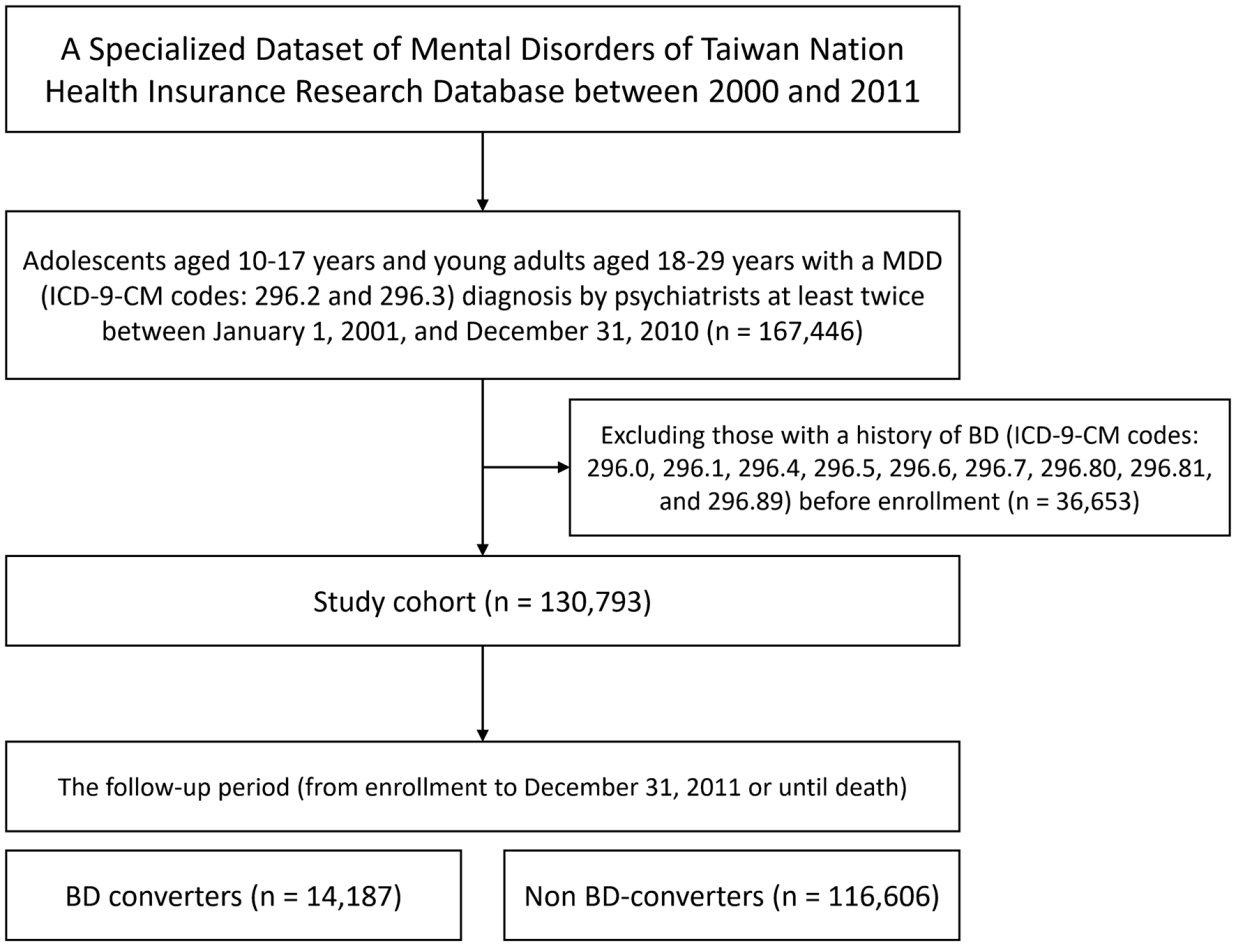
Study design

The study design is shown in the Fig. 1. We included adolescents aged 10–17 years and young adults aged 18–29 years who were diagnosed with MDD (ICD-9-CM codes: 296.2 and 296.3) by a board-certified psychiatrist at least twice between January 1, 2001, and December 31, 2010. No cases in the MDD cohort had a history of BD (ICD-9-CM codes: 296.0, 296.1, 296.4, 296.5, 296.6, 296.7, 296.80, 296.81, and 296.89) before enrollment. The time of enrollment was defined as the time of diagnosis with MDD for the first time. A diagnosis of BD during the follow-up period (from enrollment to December 31, 2011 or until death) was confirmed by at least two identifications by a board-certified psychiatrist. All of the ICD-9 codes used in the current study are provided in the Supplemental Table 1.

### Covariates

The rationales for the study variables are provided in the Supplemental Table 2. We assessed baseline psychiatric comorbidities, physical comorbidities, and family history of mental disorders. The psychiatric comorbidities included attention-deficit/hyperactivity disorder (ADHD; ICD-9-CM codes: 314.00, 314.01, 314.8, and 314.9), substance use disorder (SUD; including use of alcohol; ICD-9-CM codes: 303.0X, 303.9X, 304.0X-304.9X, 305.0X, 305.2X-305.7X, and 305.9X), smoking, posttraumatic stress disorder (PTSD; ICD-9-CM codes: 309.81), and PDs (ICD-9-CM codes: 301.0, 301.2X, 301.4, 301.5X, 301.6, 301.7, 301.8X, and 301.9). Physical comorbidities included epilepsy (ICD-9-CM codes: 345.0X–345.5X, and 345.7X–345.9X), autoimmune disease (ICD-9-CM codes: 279.0–279.5, and 279.8–279.9), any atopic disease (atopic dermatitis, allergic rhinitis, asthma, and allergic conjunctivitis; ICD-9-CM codes: 372.14, 477, 493, 691), thyroid disease (ICD-9-CM codes: 240–246), cerebrovascular disease (ICD-9-CM codes: 430–438), traumatic brain injury (TBI; ICD-9-CM codes: 850–854), and obesity. Family history of mental disorders included schizophrenia, BD, MDD, and ADHD. Antidepressant resistance was categorized into 3 groups: low, moderate, and high. An adequate trial of antidepressant resistance was defined as taking an antidepressant within its therapeutic dosage range (i.e., fluoxetine ≥ 20 mg/day) for > 60 consecutive days. On the basis of antidepressant treatment regimens and treatment response to antidepressants during the 2-year follow-up period after MDD diagnosis, participants who did not have any antidepressant prescriptions or stayed on a single antidepressant were regarded as having low antidepressant resistance (easy to treat); participants whose antidepressants were changed only once were defined as having moderate antidepressant resistance (intermediate difficulty to treat); and participants whose antidepressant treatment regimens were altered two or more times were considered to have high antidepressant resistance (treatment resistant) [25]. The Charlson Comorbidity Index (CCI) score and all-cause clinical visits were obtained for the MDD cohort. The CCI consisted of scores on 19 physical conditions and was used to assess systemic health conditions for all enrolled participants [6]. Monthly income level (≤ 15,840 New Taiwanese dollars (NTD), 15,841–25,000 NTD, and ≥ 25,000 NTD) and residential location (levels 1–5, from most to least urbanized) were regarded as proxies for healthcare availability in Taiwan [28]. Finally, the number of psychiatric visits per year was included to account for potential detection bias.

### Statistical analysis

The demographic characteristics included continuous and nominal variables. Conversion to BD was analyzed using survival analysis. The Aalen Johansen method was used to estimate conversion rates, and Cox regression was used to examine the significance of candidate predictors. Stepwise Cox regression was used to determine possible predictors of conversion among the large number of candidates. In survival analysis, the hazard ratio (HR) represented the hazard rate difference between conversion and non-conversion and thus indicated instantaneous risk over the follow-up period. A receiver-operating characteristic (ROC) curve was calculated to examine the predictive value of variables to the diagnostic conversion to BD. A two-tailed p value of less than 0.05 was considered statistically significant. All data processing and statistical analyses were performed with Statistical Analysis Software (SAS) version 9.1 (SAS Inc., Cary, NC, USA) and Statistical Package for Social Sciences (SPSS) version 22.0 (IBM Corp., Armonk, NY).

## Results

The demographic characteristics of our total sample are presented in Table 1. A total of 130,793 adolescents and young adults with MDD were enrolled (mean age: 23.38 years; female: 61.5%). During the 11-year follow-up period, 10.8% of adolescents and young adults (n = 14,187) were diagnosed with BD. Regarding antidepressant resistance, 63.7% had low antidepressant resistance, 28.7% had moderate antidepressant resistance, and 7.6% had high antidepressant resistance. The most common physical comorbidity was atopic disease (19,886; 15.2%), and the most common psychiatric comorbidity was SUD (15,899; 12.2%). The most common mental disorder in the family history was MDD (16,684; 12.8%).

**Table 1:** Demographic characteristics and conversion incidence of BD among participants with MDD

	Total sample (n = 130,793)	Converters (n = 14,187)	Non-converters (n = 116,606)
Age (years, SD)	23.38 (4.18)	23.29 (4.16)	23.40 (4.18)
Sex (n, %)			
Male	50,343 (38.5)	5445 (38.4)	44,898 (38.5)
Female	80,450 (61.5)	8762 (61.6)	71,708 (61.5)
Types of bipolar disorder (n, %)			
Bipolar I disorder		13,098 (92.3)	
Bipolar II disorder or bipolar disorder, unspecified		1089 (7.7)	
Level of antidepressant resistance (n, %)			
Easy	83,252 (63.7)	5849 (41.2)	77,403 (66.4)
Intermediate	37,544 (28.7)	5673 (40.0)	31,871 (27.3)
Resistant	9997 (7.6)	2665 (18.8)	7332 (6.3)
Physical comorbidities at baseline (n, %)			
Epilepsy	1145 (0.9)	137 (1.0)	1008 (0.9)
Autoimmune disease	878 (0.7)	77 (0.5)	801 (0.7)
Atopic disease	19,886 (15.2)	1962 (13.8)	17,924 (15.4)
Thyroid disease	2008 (1.5)	241 (1.7)	1767 (1.5)
Cerebrovascular disease	1064 (0.8)	91 (0.6)	973 (0.8)
Traumatic brain injury	15,149 (11.6)	1600 (11.3)	13,549 (11.6)
Obesity	4740 (3.6)	953 (6.7)	3787 (3.2)
CCI score (SD)	0.41 (0.74)	0.41 (0.75)	0.41 (0.74)
Psychiatric comorbidities at baseline (n, %)			
ADHD	2421 (1.9)	436 (3.1)	1985 (1.7)
Alcohol use disorder	10,703 (8.2)	2066 (14.6)	8637 (7.4)
Substance use disorder	15,899 (12.2)	3396 (23.9)	12,503 (10.7)
Smoking	7650 (5.8)	1438 (10.1)	6212 (5.3)
PTSD	1208 (0.9)	142 (1.0)	1066 (0.9)
Cluster A personality disorder	656 (0.5)	143 (1.0)	513 (0.4)
Cluster B personality disorder	9196 (7.0)	2561 (18.1)	6635 (5.7)
Cluster C personality disorder	909 (0.7)	250 (1.8)	659 (0.6)
Family history of mental disorder at baseline (n, %)			
Schizophrenia	4425 (3.4)	748 (5.3)	3677 (3.2)
Bipolar disorder	5225 (4.0)	1125 (7.9)	4100 (3.5)
Major depressive disorder	16,684 (12.8)	2425 (17.1)	14,259 (12.2)
ADHD	4178 (3.2)	582 (4.1)	3596 (3.1)
Level of urbanization (n, %)			
1 (most urbanized)	36,537 (27.9)	3787 (26.7)	32,750 (28.0)
2	45,011 (34.4)	5024 (35.3)	39,987 (34.3)
3	17,827 (13.6)	1884 (13.3)	15,943 (13.7)
4	12,679 (9.7)	1412 (10.0)	11,267 (9.7)
5 (most rural)	18,739 (14.4)	2080 (14.7)	16,659 (14.3)
Income-related insured amount (n, %)			
< 19,100 NTD/month	55,836 (42.7)	7117 (50.2)	48,719 (41.8)
19,100–42,000 NTD/month	46,352 (35.4)	4848 (34.2)	41,504 (35.6)
> 42,000 NTD/month	28,605 (21.9)	2222 (15.7)	26,383 (22.6)
Mental health visits to the clinic (times per year, SD)	4.80 (6.41)	10.90 (9.15)	4.05 (5.56)

*MDD* major depressive disorder, *BD* bipolar disorder, *SD* standard deviation, *NTD* New Taiwan dollar, *ADHD* attention-deficit/hyperactivity disorder, *PTSD* posttraumatic stress disorder, *CCI* Charlson Comorbidity Index

The conversion rate from MDD to BD over 11 years is shown in Table 2. The overall conversion rate was 13.80% (95% confidence interval (CI) 13.54–14.06%); the conversion rate was the highest in the first year (4.50%; 4.39–4.61%) and declined with time. The relationship between the rate of conversion to BD and time thus exhibited a quadratic curve. The annual conversion rate decreased after the first year, and the 3-year, 5-year, and 10-year cumulative conversion rates were 7.74% (7.59–7.88%), 9.81% (9.64–9.98%), and 13.37% (13.14–13.61%), respectively. Among the conversions, 41.8% occurred within the first year. One-third of conversions occurred within the first three years, and more than 90% of conversions occurred within the first six years.

**Table 2:** Conversion rate from MDD to BD

Time from baseline (years)	Estimated conversion rate (%, 95% CI)	Difference in annual conversion rate (%)	Cumulative number of converters (%)	Difference in annual converters (%)
1	4.50 (4.39–4.61)	4.50	5885 (41.48%)	–
2	6.31 (6.18–6.45)	1.81	8186 (57.70%)	2301 (16.22%)
3	7.74 (7.59–7.88)	1.43	9859 (9.49%)	1673 (11.79%)
4	8.86 (8.70–9.02)	1.12	11,083 (78.12%)	1224 (8.63%)
5	9.81 (9.64–9.98)	0.95	12,029 (84.79%)	946 (6.67%)
6	10.68 (10.51–10.86)	0.87	12,795 (90.19%)	766 (5.40%)
7	11.39 (11.21–11.58)	0.71	13,315 (93.85%)	520 (3.67%)
8	12.12 (11.92–12.31)	0.73	13,725 (96.74%)	410 (2.89%)
9	12.74 (12.53–12.95)	0.62	13,974 (98.50%)	249 (1.76%)
10	13.37 (13.14–13.61)	0.63	14,137 (99.65%)	163 (1.15%)
≥ 10	13.80 (13.54–14.06)	0.43	14,187 (100.00%)	50 (0.35%)


*MDD* major depressive disorder, *BD* bipolar disorder, *CI* confidence interval

Table 3 shows the factors that were most or less predictive of conversion to BD among children and adolescents with MDD according to Cox regression analysis. The risk of conversion to BD was higher among the following factors: moderate (HR: 1.81; 95% CI 1.74–1.88) and high antidepressant resistance (2.19; 2.08–2.31); obesity (1.44; 1.35–1.54); ADHD (1.32; 1.20–1.46); alcohol use disorder (1.37; 1.31–1.44); SUD (1.30; 1.23–1.37); cluster B PDs (1.88; 1.79–1.96); cluster C PDs (1.43; 1.26–1.63); a family history of schizophrenia (1.23; 1.14–1.33), BD (1.93; 1.81–2.07), and MDD (1.07; 1.02–1.12); and frequency of mental health visits to the clinic (1.04; 1.04–1.05). The risk of conversion to BD was lower among the following factors: increased age (0.99; 0.98–0.99), epilepsy (0.53; 0.44–0.64), cerebrovascular disease (0.81; 0.65–0.99), TBI (0.94; 0.89–0.99), level 3 urbanization (0.92; 0.87–0.97), and higher monthly income (15,841–25,000 NTD: 0.92; 0.89–0.96; ≥ 25,001 NTD: 0.72; 0.69–0.76).

**Table 3:** Cox regression analysis of conversion to BD among participants with MDD

	HR	95% CI	*p* value	Relative risk of conversion (↑ or ↓)
Age	**0.99**	**0.98–0.99**	**< 0.001**	↓
Male versus female (ref.)	0.99	0.96–1.02	0.541	
Level of antidepressant resistance				
Easy	1 (ref.)	–	–	
Intermediate	**1.81**	**1.74–1.88**	**< 0.001**	↑
Resistant	**2.19**	**2.08–2.31**	**< 0.001**	↑
Physical comorbidities at baseline				
Epilepsy	**0.53**	**0.44–0.64**	**< 0.001**	↓
Autoimmune disease	0.82	0.66–1.03	0.089	
Atopic disease	0.97	0.92–1.02	0.203	
Thyroid disease	1.12	0.99–1.27	0.084	
Cerebrovascular disease	**0.81**	**0.65–0.99**	**0.041**	↓
Traumatic brain injury	**0.94**	**0.89–0.99**	**0.013**	↓
Obesity	**1.44**	**1.35–1.54**	**< 0.001**	↑
CCI score	0.99	0.97–1.01	0.342	
Psychiatric comorbidities at baseline				
ADHD	**1.32**	**1.20–1.46**	**< 0.001**	↑
Alcohol use disorder	**1.37**	**1.31–1.44**	**< 0.001**	↑
Substance use disorder	**1.30**	**1.23–1.37**	**< 0.001**	↑
Smoking	1.04	0.97–1.11	0.301	
PTSD	1.01	0.85–1.19	0.954	
Cluster A personality disorder	0.90	0.76–1.07	0.245	
Cluster B personality disorder	**1.88**	**1.79–1.96**	**< 0.001**	↑
Cluster C personality disorder	**1.43**	**1.26–1.63**	**< 0.001**	↑
Family history of mental disorder at baseline				
Schizophrenia	**1.23**	**1.14–1.33**	**< 0.001**	↑
Bipolar disorder	**1.93**	**1.81–2.07**	**< 0.001**	↑
Major depressive disorder	**1.07**	**1.02–1.12**	**0.007**	↑
ADHD	1.07	0.99–1.17	0.095	
Level of urbanization				
1 (most urbanized)	1 (ref.)	–	–	
2	1.02	0.98–1.07	0.345	
3	**0.92**	**0.87–0.97**	**0.002**	↓
4	1.02	0.96–1.08	0.560	
5 (most rural)	1.00	0.95–1.05	0.958	
Income-related insured amount				
≤ 15,840 NTD/month	1 (ref.)	–	–	
15,841–25,000 NTD/month	**0.92**	**0.89–0.96**	**< 0.001**	↓
≥ 25,001 NTD/month	**0.72**	**0.69–0.76**	**< 0.001**	↓
Mental health visits to the clinic per year	**1.04**	**1.04–1.05**	**< 0.001**	↑

*MDD* major depressive disorder, *BD* bipolar disorder, *HR* hazard ratio, *CI* confidence interval, *NTD* New Taiwan dollar, *ADHD* attention-deficit/hyperactivity disorder, *PTSD* posttraumatic stress disorder, *CCI* Charlson Comorbidity Index

Bold type indicates statistical significance

**Fig. 2:**
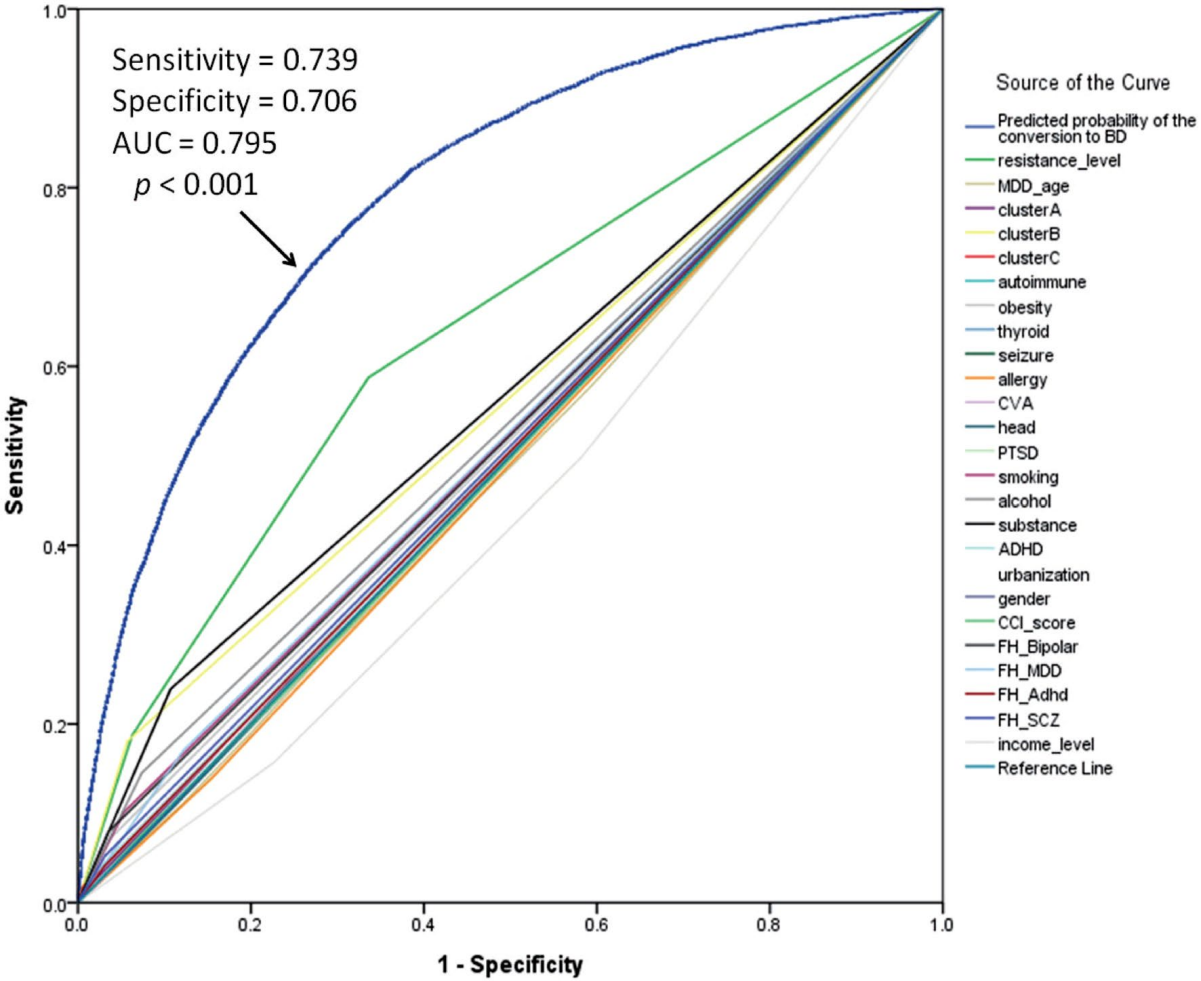
ROC curve of the predicted probability of conversion from MDD to BD. *ROC* receiver-operator characteristic, *MDD* major depressive disorder, *BD* bipolar disorder, *AUC* area under the curve

Figure 2 presents the predictive value of each factor. A composite of demographic characteristics, antidepressant resistance, physical and psychiatric comorbidities, and family history of mental disorders significantly predicted conversion from MDD to BD (area under the curve (AUC) = 0.795, *p* < 0.001), indicating significant and acceptable discrimination for BD conversion among participants with MDD.

## Discussion

We found that the risk of conversion to BD was higher among individuals with younger age of diagnosis with MDD, moderate or high antidepressant resistance, obesity, psychiatric comorbidities (ADHD, alcohol use disorder, SUD, cluster B PDs and cluster C PDs), family history of mental disorders (schizophrenia, BD, and MDD), lower monthly income, and more mental health visits to the clinic each year. In contrast, the risk of conversion to BD was lower among individuals with the physical comorbidities of epilepsy, cerebrovascular disease, and TBI.

In our study, the conversion rate from MDD to BD among adolescents and young adults was 13.80% over the observation period (11 years), which is almost double that reported in a previous nationwide study in Korea [24]. However, the age range of our sample was broader (including adolescents) than that of the Korean study, which only included young adults. In addition, the average follow-up period of the Korean study was 3.26 years. Previous studies have concluded that the risk of BD conversion has a linear relationship with time [1, 10, 14]. However, these studies had relatively small sample sizes (ranging from 74 to 309 individuals). We used a nationwide dataset and found that the risk was highest in the first year after MDD diagnosis and decreased in subsequent years, representing a quadratic (rather than linear) relationship.

For adolescents and young adults diagnosed with MDD, the BD conversion rate was the highest in the first year and declined with time. In addition to intensive follow-up and greater participant retention [36], we considered that age and treatment resistance may contributed to this finding, while the potential mechanisms are complex. A systematic review reported that younger age of onset is a risk factor of treatment-resistance [34]. Therefore, our study population may be more vulnerable to treatment resistance than the adult population. This also explained the decreasing trend of diagnostic conversion with time. Besides, Danish [33] and Korean [23] nationwide cohort studies reported that the conversion rate plummeted between the first and second years, and then decreased to a lesser extent in subsequent years. Our cumulative conversion rate is higher than the Danish data, probably because we focused on adolescents and young adults; these ages represent the times of peak conversion. Interestingly, another national cohort study in England found a conversion rate that followed a linear function with age in individuals 15–35 years old [19]. This study observed a peak age of conversion of 30–35 years old, which is inconsistent with the peak age of 20.5 years old from a worldwide large-scale meta-analysis of 192 epidemiological studies [40]. The mismatch could derive from the significantly higher conversion rate in females compared to males, as the English study had a preponderance of female participants; as found in a English study about sex differences in age at onset of BD covering a 35-year period [22], females have higher incidence rates of BD during age bands from 26 to 75 years old, despite the sex difference not reaching statistical significance in individual age bands. We found that the estimated conversion rate exhibited a tendency of gradually slowing growth, accompanied by a continuous rise in the cumulative number of conversions, albeit to a lesser extent.

Obesity and personality disorder have not been considered as risk factors of BD conversion in previous studies addressing the adult population. Previous studies have suggested a bidirectional relationship between obesity and mood disorders in adults [29, 39], and the National Comorbidity Survey Replication reported that people with obesity had 1.47-fold greater odds of lifetime BD [37, 39]. TCF7L2 has been identified as a risk allele associated with BD susceptibility and higher body mass index (BMI), [44] which may explain this relationship. Furthermore, obesity has been proposed to lead to antidepressant resistance in people with MDD [42] and aggravate the severity of MDD, which increases the risk of BD [33]. On the other hand, our study also found that cluster C PDs were associated with BD conversion, an association that has not previously been examined. Previous studies have reported that obsessive–compulsive PD is the most frequently linked to BD [5, 38]; this disorder falls within cluster C PDs, which may explain our findings.

The literature has suggested a family association of BD with SUD [27], suggesting they may have a genetic relationship. Another study proposed that intermittent stressors could be a precipitating factor for both BD and SUD, as they exhibited cross sensitization [35]. Affective instability, mood dysregulation, and impaired impulse control are prominent factors in the development of BD [31].

Baseline physical comorbidities of epilepsy, cerebrovascular disease and TBI were negatively associated with BD. The neuropathological changes induced by TBI might result in deactivation of the dorsolateral prefrontal cortex and activation of ventral limbic and paralimbic structures, including the amygdala, which plays a significant role in the complex pathophysiology of MDD [20]. A meta-analysis in 2013 suggested that the prevalence of depression among individuals with epilepsy was up to 23% in some studies [11]. Both epilepsy and cerebrovascular disease have bidirectional relationships with MDD, suggesting common pathogenic mechanisms between these two physical comorbidities and MDD [16, 21]. Another possibility is related to the use of antiepileptic drugs, which are often used as mood stabilizers for the treatment of BD [4], reducing the risk of manic episodes [43].

Our study has limitations. First, our data spanned 11 years; however, diagnostic conversion from MDD to BD could still be underestimated because meta-analytic evidence has shown that conversion to BD might take, on average, 12–18 years [36]. Second, we recorded antidepressant resistance but did not explore medication types and use duration; the antidepressants administered could potentially promote the conversion to BD (e.g., tricyclic antidepressants vs. serotonin-noradrenaline reuptake inhibitors) [13]. Third, we were not able to assess genetic factors and gene‒environment interactions, although we adjusted for a family history of mental disorders at baseline. Fourth, because of long-term follow-up duration, the association between diagnostic conversion and medication type, dosage, and duration are difficult to explore. Fifth, we cannot completely confirm that all case included in the MDD cohort did not have a history of BD because some patients might have a previous manic episode without visiting medical service. Sixth, it is difficult to evaluate medication adherence in the claim database. Seventh, our study utilizing the NHI system research database did not encompass personal lifestyle factors, such as sleep habits, which could potentially influence the risk of bipolar disorder. This omission should be considered a limitation. Finally, while we discovered an association between obesity and the risk of converting to BD, individuals with MDD might receive mood stabilizers if they don't respond adequately to antidepressant treatment. In cases where weight gain occurs as a side effect of mood stabilizers, and the diagnosis is subsequently changed to BD, it's possible that obesity could be inaccurately linked to a risk factor for BD conversion.

In conclusion, we found several factors related to the diagnostic conversion from MDD to BD in adolescent and young adult populations that have not previously been investigated. These factors that were most predictive of conversion included younger age of diagnosis with MDD, moderate or high antidepressant resistance, obesity, psychiatric comorbidities (ADHD, SUD (especially alcohol), and cluster B and C PDs), family history of mental disorders (schizophrenia and mood disorder), lower monthly income, and more mental health visits to the clinic each year. In contrast, risk of conversion to BD was lower among individuals with epilepsy, cerebrovascular disease, and TBI. Because pediatric BD is usually characterized by resistance to treatment, a chronic course, and worse outcomes with impaired function [12, 15], our study findings are important as they may facilitate early screening and intervention for patients with MDD at higher risk of BD. Further research is needed to clarify the mechanisms underlying diagnostic conversion from MDD to BD.

## Supplementary Information

Below is the link to the electronic supplementary material.

Supplementary file1 (DOCX 58 KB)

Supplementary file2 (DOCX 34 KB)

## Data Availability

The NHIRD was released and audited by the Department of Health and Bureau of the National Health Insurance Program for the purpose of scientific research (https://nhird.nhri.org.tw/). Access to the NHIRD can be obtained through formal application.
