# Supplementary material for: Diagnostic conversion to bipolar disorder among adolescents and young adults with major depressive disorder: a nationwide longitudinal study
Source: Eur Child Adolesc Psychiatry. 2024 Mar 29;33(10):3625–35. doi: 10.1007/s00787-024-02401-1 (PMC11564236; doi:10.1007/s00787-024-02401-1)
Supplement: Supplementary file 1 — Supplementary file1 (DOCX 58 KB) [file 787_2024_2401_MOESM1_ESM.docx]

**Supplementary Table 1. Bipolar disorder, psychiatric comorbidities, and physical comorbidities on the International Classification of Diseases, 9th Revision (ICD-9)**

| **296** | **Affective Psychoses** |
| --- | --- |
| 296.0 | Bipolar I disorder, single manic episode |
| 296.00 | Bipolar I disorder, single manic episode, unspecified |
| 296.01 | Bipolar I disorder, single manic episode, mild |
| 296.02 | Bipolar I disorder, single manic episode, moderate |
| 296.03 | Bipolar I disorder, single manic episode, severe, without mention of psychotic behavior |
| 296.04 | Bipolar I disorder, single manic episode, severe, specified as with psychotic behavior |
| 296.05 | Bipolar I disorder, single manic episode, in partial or unspecified remission |
| 296.06 | Bipolar I disorder, single manic episode, in full remission |
| 296.1 | Manic disorder recurrent episode |
| 296.10 | Manic affective disorder, recurrent episode, unspecified |
| 296.11 | Manic affective disorder, recurrent episode, mild |
| 296.12 | Manic affective disorder, recurrent episode, moderate |
| 296.13 | Manic affective disorder, recurrent episode, severe, without mention of psychotic behavior |
| 296.14 | Manic affective disorder, recurrent episode, severe, specified as with psychotic behavior |
| 296.15 | Manic affective disorder, recurrent episode, in partial or unspecified remission |
| 296.16 | Manic affective disorder, recurrent episode, in full remission |
| 296.4 | Bipolar I disorder, most recent episode (or current) manic |
| 296.40 | Bipolar I disorder, most recent episode (or current) manic, unspecified |
| 296.41 | Bipolar I disorder, most recent episode (or current) manic, mild |
| 296.42 | Bipolar I disorder, most recent episode (or current) manic, moderate |
| 296.43 | Bipolar I disorder, most recent episode (or current) manic, severe, without mention of psychotic behavior |
| 296.44 | Bipolar I disorder, most recent episode (or current) manic, severe, specified as with psychotic behavior |
| 296.45 | Bipolar I disorder, most recent episode (or current) manic, in partial or unspecified remission |
| 296.46 | Bipolar I disorder, most recent episode (or current) manic, in full remission |
| 296.5 | Bipolar I disorder, most recent episode (or current) depressed |
| 296.50 | Bipolar I disorder, most recent episode (or current) depressed, unspecified |
| 296.51 | Bipolar I disorder, most recent episode (or current) depressed, mild |
| 296.52 | Bipolar I disorder, most recent episode (or current) depressed, moderate |
| 296.53 | Bipolar I disorder, most recent episode (or current) depressed, severe, without mention of psychotic behavior |
| 296.54 | Bipolar I disorder, most recent episode (or current) depressed, severe, specified as with psychotic behavior |
| 296.55 | Bipolar I disorder, most recent episode (or current) depressed, in partial or unspecified remission |
| 296.56 | Bipolar I disorder, most recent episode (or current) depressed, in full remission |
| 296.6 | Bipolar I disorder, most recent episode (or current) mixed |
| 296.60 | Bipolar I disorder, most recent episode (or current) mixed, unspecified |
| 296.61 | Bipolar I disorder, most recent episode (or current) mixed, mild |
| 296.62 | Bipolar I disorder, most recent episode (or current) mixed, moderate |
| 296.63 | Bipolar I disorder, most recent episode (or current) mixed, severe, without mention of psychotic behavior |
| 296.64 | Bipolar I disorder, most recent episode (or current) mixed, severe, specified as with psychotic behavior |
| 296.65 | Bipolar I disorder, most recent episode (or current) mixed, in partial or unspecified remission |
| 296.66 | Bipolar I disorder, most recent episode (or current) mixed, in full remission |
| 296.7 | Bipolar I disorder, most recent episode (or current) unspecified |
| 296.8 | Other and unspecified bipolar disorders |
| 296.80 | Bipolar disorder, unspecified |
| 296.81 | Atypical manic disorder |
| 296.89 | Other bipolar disorders |
| **314** | **Hyperkinetic Syndrome of Childhood** |
| 314.0 | Attention deficit disorder of childhood |
| 314.00 | Attention deficit disorder without mention of hyperactivity |
| 314.01 | Attention deficit disorder with hyperactivity |
| 314.8 | Other specified manifestations of hyperkinetic syndrome |
| 314.9 | Unspecified hyperkinetic syndrome (Include: hyperkinetic reaction of childhood or adolescence NOS, hyperkinetic syndrome NOS) |
| **303-305** | **Psychoactive Substance** |
| 303.0 | Acute alcoholic intoxication (Include: acute drunkenness in alcoholism, dipsomania, chronic alcoholism) |
| 303.00 | Acute alcoholic intoxication in alcoholism, unspecified |
| 303.01 | Acute alcoholic intoxication in alcoholism, continuous |
| 303.02 | Acute alcoholic intoxication in alcoholism, episodic |
| 303.03 | Acute alcoholic intoxication in alcoholism, in remission |
| 303.9 | Other and unspecified alcohol dependence |
| 303.90 | Other and unspecified alcohol dependence, unspecified |
| 303.91 | Other and unspecified alcohol dependence, continuous |
| 303.92 | Other and unspecified alcohol dependence, episodic |
| 303.93 | Other and unspecified alcohol dependence, in remission |
| 304.0 | Opioid type dependence (Include drugs: heroin, methadone, opium, opium alkaloids and their derivatives, synthetics with morphine-like effects) |
| 304.00 | Opioid type dependence, unspecified |
| 304.01 | Opioid type dependence, continuous |
| 304.02 | Opioid type dependence, episodic |
| 304.03 | Opioid type dependence, in remission |
| 304.1 | Sedative, hypnotic or anxiolytic dependence |
| 304.10 | Sedative, hypnotic or anxiolytic dependence, unspecified |
| 304.11 | Sedative, hypnotic or anxiolytic dependence, continuous |
| 304.12 | Sedative, hypnotic or anxiolytic dependence, episodic |
| 304.13 | Sedative, hypnotic or anxiolytic dependence, in remission |
| 304.2 | Cocaine dependence |
| 304.20 | Cocaine dependence, unspecified |
| 304.21 | Cocaine dependence, continuous |
| 304.22 | Cocaine dependence, episodic |
| 304.23 | Cocaine dependence, in remission |
| 304.3 | Cannabis dependence |
| 304.30 | Cannabis dependence, unspecified |
| 304.31 | Cannabis dependence, continuous |
| 304.32 | Cannabis dependence, episodic |
| 304.33 | Cannabis dependence, in remission |
| 304.4 | Amphetamine and other psychostimulant dependence (Include drugs: phenmetrazine, methylphenidate) |
| 304.40 | Amphetamine and other psychostimulant dependence, unspecified |
| 304.41 | Amphetamine and other psychostimulant dependence, continuous |
| 304.42 | Amphetamine and other psychostimulant dependence, episodic |
| 304.43 | Amphetamine and other psychostimulant dependence, in remission |
| 304.5 | Hallucinogen dependence (Include drugs: LSD and derivatives, mescaline, psilocybin) |
| 304.50 | Hallucinogen dependence, unspecified |
| 304.51 | Hallucinogen dependence, continuous |
| 304.52 | Hallucinogen dependence, episodic |
| 304.53 | Hallucinogen dependence, in remission |
| 304.6 | Other specified drug dependence (Include: absinthe addiction, glue sniffing) |
| 304.60 | Other specified drug dependence, unspecified |
| 304.61 | Other specified drug dependence, continuous |
| 304.62 | Other specified drug dependence, episodic |
| 304.63 | Other specified drug dependence, in remission |
| 304.7 | Combinations of opioid type drug with any other drug dependence |
| 304.70 | Combinations of opioid type drug with any other drug dependence, unspecified |
| 304.71 | Combinations of opioid type drug with any other drug dependence, continuous |
| 304.72 | Combinations of opioid type drug with any other drug dependence, episodic |
| 304.73 | Combinations of opioid type drug with any other drug dependence, in remission |
| 304.8 | Combinations of drug dependence excluding opioid type drug |
| 304.80 | Combinations of drug dependence excluding opioid type drug, unspecified |
| 304.81 | Combinations of drug dependence excluding opioid type drug, continuous |
| 304.82 | Combinations of drug dependence excluding opioid type drug, episodic |
| 304.83 | Combinations of drug dependence excluding opioid type drug, in remission |
| 304.9 | Unspecified drug dependence (Include: drug addiction NOS, drug dependence NOS) |
| 304.90 | Unspecified drug dependence, unspecified |
| 304.91 | Unspecified drug dependence, continuous |
| 304.92 | Unspecified drug dependence, episodic |
| 304.93 | Unspecified drug dependence, in remission |
| 305.0 | Nondependent alcohol abuse (Include: Drunkenness NOS; Excessive drinking of alcohol NOS; "Hangover" (alcohol); Inebriety NOS) |
| 305.00 | Alcohol abuse, unspecified |
| 305.01 | Alcohol abuse, continuous |
| 305.02 | Alcohol abuse, episodic |
| 305.03 | Alcohol abuse, in remission |
| 305.2 | Nondependent cannabis abuse |
| 305.20 | Cannabis abuse, unspecified |
| 305.21 | Cannabis abuse, continuous |
| 305.22 | Cannabis abuse, episodic |
| 305.23 | Cannabis abuse, in remission |
| 305.3 | Nondependent hallucinogen abuse |
| 305.30 | Hallucinogen abuse, unspecified |
| 305.31 | Hallucinogen abuse, continuous |
| 305.32 | Hallucinogen abuse, episodic |
| 305.33 | Hallucinogen abuse, in remission |
| 305.4 | Nondependent sedative, hypnotic or anxiolytic abuse |
| 305.40 | Sedative, hypnotic or anxiolytic abuse, unspecified |
| 305.41 | Sedative, hypnotic or anxiolytic abuse, continuous |
| 305.42 | Sedative, hypnotic or anxiolytic abuse, episodic |
| 305.43 | Sedative, hypnotic or anxiolytic abuse, in remission |
| 305.5 | Nondependent opioid abuse |
| 305.50 | Opioid abuse, unspecified |
| 305.51 | Opioid abuse, continuous |
| 305.52 | Opioid abuse, episodic |
| 305.53 | Opioid abuse, in remission |
| 305.6 | Nondependent cocaine abuse |
| 305.60 | Cocaine abuse, unspecified |
| 305.61 | Cocaine abuse, continuous |
| 305.62 | Cocaine abuse, episodic |
| 305.63 | Cocaine abuse, in remission |
| 305.7 | Nondependent amphetamine or related acting sympathomimetic abuse |
| 305.70 | Amphetamine or related acting sympathomimetic abuse, unspecified |
| 305.71 | Amphetamine or related acting sympathomimetic abuse, continuous |
| 305.72 | Amphetamine or related acting sympathomimetic abuse, episodic |
| 305.73 | Amphetamine or related acting sympathomimetic abuse, in remission |
| 305.9 | Nondependent other mixed or unspecified drug abuse |
| 305.90 | Other, mixed, or unspecified drug abuse, unspecified |
| 305.91 | Other, mixed, or unspecified drug abuse, continuous |
| 305.92 | Other, mixed, or unspecified drug abuse, episodic |
| 305.93 | Other, mixed, or unspecified drug abuse, in remission |
|  | **Posttraumatic stress disorder** |
| 309.81 | Posttraumatic stress disorder |
| **301** | **Personality disorders** |
| 301.0 | Paranoid personality disorder |
| 301.2 | Schizoid personality disorder |
| 301.20 | Schizoid personality disorder, unspecified |
| 301.21 | Introverted personality |
| 301.22 | Schizotypal personality disorder |
| 301.4 | Obsessive-compulsive personality disorder |
| 301.5 | Histrionic personality disorder |
| 301.50 | Histrionic personality disorder, unspecified |
| 301.51 | Chronic factitious illness with physical symptoms |
| 301.59 | Other histrionic personality disorder |
| 301.6 | Dependent personality disorder |
| 301.7 | Antisocial personality disorder |
| 301.8 | Other personality disorders |
| 301.81 | Narcissistic personality disorder |
| 301.82 | Avoidant personality disorder |
| 301.83 | Borderline personality disorder |
| 301.84 | Passive-aggressive personality |
| 301.89 | Other personality disorders |
| 301.9 | Unspecified personality disorder |
| **345** | **Epilepsy and recurrent seizures** |
| 345.0 | Generalized nonconvulsive epilepsy |
| 345.00 | Generalized nonconvulsive epilepsy, without mention of intractable epilepsy |
| 345.01 | Generalized nonconvulsive epilepsy, with intractable epilepsy |
| 345.1 | Generalized convulsive epilepsy |
| 345.10 | Generalized convulsive epilepsy, without mention of intractable epilepsy |
| 345.11 | Generalized convulsive epilepsy, with intractable epilepsy |
| 345.2 | Petit mal status |
| 345.3 | Grand mal status |
| 345.4 | Localization-related (focal) (partial) epilepsy and epileptic syndromes with complex partial seizures |
| 345.40 | Localization-related (focal) (partial) epilepsy and epileptic syndromes with complex partial seizures, without mention of intractable epilepsy |
| 345.41 | Localization-related (focal) (partial) epilepsy and epileptic syndromes with complex partial seizures, with intractable epilepsy |
| 345.5 | Localization-related (focal) (partial) epilepsy and epileptic syndromes with simple partial seizures |
| 345.50 | Localization-related (focal) (partial) epilepsy and epileptic syndromes with simple partial seizures, without mention of intractable epilepsy |
| 345.51 | Localization-related (focal) (partial) epilepsy and epileptic syndromes with simple partial seizures, with intractable epilepsy |
| 345.7 | Epilepsia partialis continua |
| 345.70 | Epilepsia partialis continua, without mention of intractable epilepsy |
| 345.71 | Epilepsia partialis continua, with intractable epilepsy |
| 345.8 | Other forms of epilepsy and recurrent seizures |
| 345.80 | Other forms of epilepsy and recurrent seizures, without mention of intractable epilepsy |
| 345.81 | Other forms of epilepsy and recurrent seizures, with intractable epilepsy |
| 345.9 | Epilepsy unspecified |
| 345.90 | Epilepsy, unspecified, without mention of intractable epilepsy |
| 345.91 | Epilepsy, unspecified, with intractable epilepsy |
| **279** | **Disorders involving the immune mechanism** |
| 279.0 | Deficiency of humoral immunity |
| 279.00 | Hypogammaglobulinemia, unspecified |
| 279.01 | Selective IgA immunodeficiency |
| 279.02 | Selective IgM immunodeficiency |
| 279.03 | Other selective immunoglobulin deficiencies |
| 279.04 | Congenital hypogammaglobulinemia |
| 279.05 | Immunodeficiency with increased IgM |
| 279.06 | Common variable immunodeficiency |
| 279.09 | Other deficiency of humoral immunity |
| 279.1 | Deficiency of cell-mediated immunity |
| 279.10 | Immunodeficiency with predominant T-cell defect, unspecified |
| 279.11 | Digeorge's syndrome |
| 279.12 | Wiskott-aldrich syndrome |
| 279.13 | Nezelof's syndrome |
| 279.19 | Other deficiency of cell-mediated immunity |
| 279.2 | Combined immunity deficiency |
| 279.3 | Unspecified immunity deficiency |
| 279.4 | Autoimmune disease not elsewhere classified |
| 279.41 | Autoimmune lymphoproliferative syndrome |
| 279.49 | Autoimmune disease, not elsewhere classified |
| 279.5 | Graft-versus-host disease |
| 279.50 | Graft-versus-host disease, unspecified |
| 279.51 | Acute graft-versus-host disease |
| 279.52 | Chronic graft-versus-host disease |
| 279.53 | Acute on chronic graft-versus-host disease |
| 279.8 | Other specified disorders involving the immune mechanism |
| 279.9 | Unspecified disorder of immune mechanism |
| **477** | **Allergic rhinitis** |
| 477.0 | Allergic rhinitis due to pollen |
| 477.1 | Allergic rhinitis due to food |
| 477.2 | Allergic rhinitis due to animal (cat) (dog) hair and dander |
| 477.8 | Allergic rhinitis due to other allergen |
| 477.9 | Allergic rhinitis, cause unspecified |
| **493** | **Asthma** |
| 493.0 | Extrinsic asthma |
| 493.00 | Extrinsic asthma, unspecified |
| 493.01 | Extrinsic asthma with status asthmaticus |
| 493.02 | Extrinsic asthma with (acute) exacerbation |
| 493.1 | Intrinsic asthma |
| 493.10 | Intrinsic asthma, unspecified |
| 493.11 | Intrinsic asthma with status asthmaticus |
| 493.12 | Intrinsic asthma with (acute) exacerbation |
| 493.2 | Chronic obstructive asthma |
| 493.20 | Chronic obstructive asthma, unspecified |
| 493.21 | Chronic obstructive asthma with status asthmaticus |
| 493.22 | Chronic obstructive asthma with (acute) exacerbation |
| 493.8 | Other forms of asthma |
| 493.81 | Exercise induced bronchospasm |
| 493.82 | Cough variant asthma |
| 493.9 | Asthma unspecified |
| 493.90 | Asthma,unspecified type, unspecified |
| 493.91 | Asthma, unspecified type, with status asthmaticus |
| 493.92 | Asthma, unspecified type, with (acute) exacerbation |
| **691** | **Atopic dermatitis and related conditions** |
| 691.0 | Diaper or napkin rash |
| 691.8 | Other atopic dermatitis and related conditions |
|  | **Other atopic disease** |
| 372.14 | Other chronic allergic conjunctivitis |
| **240-246** | **Disorders of thyroid gland** |
| 240.0 | Goiter, specified as simple |
| 240.9 | Goiter, unspecified |
| 241.0 | Nontoxic uninodular goiter |
| 241.1 | Nontoxic multinodular goiter |
| 241.9 | Unspecified nontoxic nodular goiter |
| 242.0 | Toxic diffuse goiter |
| 242.00 | Toxic diffuse goiter without mention of thyrotoxic crisis or storm |
| 242.01 | Toxic diffuse goiter with mention of thyrotoxic crisis or storm |
| 242.1 | Toxic uninodular goiter |
| 242.10 | Toxic uninodular goiter without mention of thyrotoxic crisis or storm |
| 242.11 | Toxic uninodular goiter with mention of thyrotoxic crisis or storm |
| 242.2 | Toxic multinodular goiter |
| 242.20 | Toxic multinodular goiter without mention of thyrotoxic crisis or storm |
| 242.21 | Toxic multinodular goiter with mention of thyrotoxic crisis or storm |
| 242.3 | Toxic nodular goiter unspecified type |
| 242.30 | Toxic nodular goiter, unspecified type, without mention of thyrotoxic crisis or storm |
| 242.31 | Toxic nodular goiter, unspecified type, with mention of thyrotoxic crisis or storm |
| 242.4 | Thyrotoxicosis from ectopic thyroid nodule |
| 242.40 | Thyrotoxicosis from ectopic thyroid nodule without mention of thyrotoxic crisis or storm |
| 242.41 | Thyrotoxicosis from ectopic thyroid nodule with mention of thyrotoxic crisis or storm |
| 242.8 | Thyrotoxicosis of other specified origin |
| 242.80 | Thyrotoxicosis of other specified origin without mention of thyrotoxic crisis or storm |
| 242.81 | Thyrotoxicosis of other specified origin with mention of thyrotoxic crisis or storm |
| 242.9 | Thyrotoxicosis without mention of goiter or other cause |
| 242.90 | Thyrotoxicosis without mention of goiter or other cause, and without mention of thyrotoxic crisis or storm |
| 242.91 | Thyrotoxicosis without mention of goiter or other cause, with mention of thyrotoxic crisis or storm |
| 243 | Congenital hypothyroidism |
| 244.0 | Post-surgical hypothyroidism |
| 244.1 | Other post-ablative hypothyroidism |
| 244.2 | Iodine hypothyroidism |
| 244.3 | Other iatrogenic hypothyroidism |
| 244.8 | Other specified acquired hypothyroidism |
| 244.9 | Unspecified acquired hypothyroidism |
| 245.0 | Acute thyroiditis |
| 245.1 | Subacute thyroiditis |
| 245.2 | Chronic lymphocytic thyroiditis |
| 245.3 | Chronic fibrous thyroiditis |
| 245.4 | Iatrogenic thyroiditis |
| 245.8 | Other and unspecified chronic thyroiditis |
| 245.9 | Thyroiditis, unspecified |
| 246.0 | Disorders of thyrocalcitonin secretion |
| 246.1 | Dyshormonogenic goiter |
| 246.2 | Cyst of thyroid |
| 246.3 | Hemorrhage and infarction of thyroid |
| 246.8 | Other specified disorders of thyroid |
| 246.9 | Unspecified disorder of thyroid |
| **430-438** | **Cerebrovascular disease** |
| 430 | Subarachnoid hemorrhage |
| 431 | Intracerebral hemorrhage |
| 432.0 | Nontraumatic extradural hemorrhage |
| 432.1 | Subdural hemorrhage |
| 432.9 | Unspecified intracranial hemorrhage |
| 433.0 | Occlusion and stenosis of basilar artery |
| 433.00 | Occlusion and stenosis of basilar artery without mention of cerebral infarction |
| 433.01 | Occlusion and stenosis of basilar artery with cerebral infarction |
| 433.1 | Occlusion and stenosis of carotid artery |
| 433.10 | Occlusion and stenosis of carotid artery without mention of cerebral infarction |
| 433.11 | Occlusion and stenosis of carotid artery with cerebral infarction |
| 433.2 | Occlusion and stenosis of vertebral artery |
| 433.20 | Occlusion and stenosis of vertebral artery without mention of cerebral infarction |
| 433.21 | Occlusion and stenosis of vertebral artery with cerebral infarction |
| 433.3 | Occlusion and stenosis of multiple and bilateral precerebral arteries |
| 433.30 | Occlusion and stenosis of multiple and bilateral precerebral arteries without mention of cerebral infarction |
| 433.31 | Occlusion and stenosis of multiple and bilateral precerebral arteries with cerebral infarction |
| 433.8 | Occlusion and stenosis of other specified precerebral artery |
| 433.80 | Occlusion and stenosis of other specified precerebral artery without mention of cerebral infarction |
| 433.81 | Occlusion and stenosis of other specified precerebral artery with cerebral infarction |
| 433.9 | Occlusion and stenosis of unspecified precerebral artery |
| 433.90 | Occlusion and stenosis of unspecified precerebral artery without mention of cerebral infarction |
| 433.91 | Occlusion and stenosis of unspecified precerebral artery with cerebral infarction |
| 434.0 | Cerebral thrombosis |
| 434.00 | Cerebral thrombosis without mention of cerebral infarction |
| 434.01 | Cerebral thrombosis with cerebral infarction |
| 434.1 | Cerebral embolism |
| 434.10 | Cerebral embolism without mention of cerebral infarction |
| 434.11 | Cerebral embolism with cerebral infarction |
| 434.9 | Cerebral artery occlusion unspecified |
| 434.90 | Cerebral artery occlusion, unspecified without mention of cerebral infarction |
| 434.91 | Cerebral artery occlusion, unspecified with cerebral infarction |
| 435.0 | Basilar artery syndrome |
| 435.1 | Vertebral artery syndrome |
| 435.2 | Subclavian steal syndrome |
| 435.3 | Vertebrobasilar artery syndrome |
| 435.8 | Other specified transient cerebral ischemias |
| 435.9 | Unspecified transient cerebral ischemia |
| 436 | Acute, but ill-defined, cerebrovascular disease |
| 437.0 | Cerebral atherosclerosis |
| 437.1 | Other generalized ischemic cerebrovascular disease |
| 437.2 | Hypertensive encephalopathy |
| 437.3 | Cerebral aneurysm, nonruptured |
| 437.4 | Cerebral arteritis |
| 437.5 | Moyamoya disease |
| 437.6 | Nonpyogenic thrombosis of intracranial venous sinus |
| 437.7 | Transient global amnesia |
| 437.8 | Other ill-defined cerebrovascular disease |
| 437.9 | Unspecified cerebrovascular disease |
| 438.0 | Late effects of cerebrovascular disease, cognitive deficits |
| 438.1 | Speech and language deficits |
| 438.10 | Late effects of cerebrovascular disease, speech and language deficit, unspecified |
| 438.11 | Late effects of cerebrovascular disease, aphasia |
| 438.12 | Late effects of cerebrovascular disease, dysphasia |
| 438.13 | Late effects of cerebrovascular disease, dysarthria |
| 438.14 | Late effects of cerebrovascular disease, fluency disorder |
| 438.19 | Late effects of cerebrovascular disease, other speech and language deficits |
| 438.2 | Hemiplegia/hemiparesis |
| 438.20 | Late effects of cerebrovascular disease, hemiplegia affecting unspecified side |
| 438.21 | Late effects of cerebrovascular disease, hemiplegia affecting dominant side |
| 438.22 | Late effects of cerebrovascular disease, hemiplegia affecting nondominant side |
| 438.3 | Monoplegia of upper limb |
| 438.30 | Late effects of cerebrovascular disease, monoplegia of upper limb affecting unspecified side |
| 438.31 | Late effects of cerebrovascular disease, monoplegia of upper limb affecting dominant side |
| 438.32 | Late effects of cerebrovascular disease, monoplegia of upper limb affecting nondominant side |
| 438.4 | Monoplegia of lower limb |
| 438.40 | Late effects of cerebrovascular disease, monoplegia of lower limb affecting unspecified side |
| 438.41 | Late effects of cerebrovascular disease, monoplegia of lower limb affecting dominant side |
| 438.42 | Late effects of cerebrovascular disease, monoplegia of lower limb affecting nondominant side |
| 438.5 | Other paralytic syndrome |
| 438.50 | Late effects of cerebrovascular disease, other paralytic syndrome affecting unspecified side |
| 438.51 | Late effects of cerebrovascular disease, other paralytic syndrome affecting dominant side |
| 438.52 | Late effects of cerebrovascular disease, other paralytic syndrome affecting nondominant side |
| 438.53 | Late effects of cerebrovascular disease, other paralytic syndrome, bilateral |
| 438.6 | Late effects of cerebrovascular disease, alterations of sensations |
| 438.7 | Late effects of cerebrovascular disease, disturbances of vision |
| 438.8 | Other late effects of cerebrovascular disease |
| 438.81 | Other late effects of cerebrovascular disease, apraxia |
| 438.82 | Other late effects of cerebrovascular disease, dysphagia |
| 438.83 | Other late effects of cerebrovascular disease, facial weakness |
| 438.84 | Other late effects of cerebrovascular disease, ataxia |
| 438.85 | Other late effects of cerebrovascular disease, vertigo |
| 438.89 | Other late effects of cerebrovascular disease |
| 438.9 | Unspecified late effects of cerebrovascular disease |
| **850-854** | **Intracranial injury, excluding those with skull fracture** |
| 850.0 | Concussion with no loss of consciousness |
| 850.1 | Concussion with brief loss of consciousness |
| 850.11 | Concussion, with loss of consciousness of 30 minutes or less |
| 850.12 | Concussion, with loss of consciousness from 31 to 59 minutes |
| 850.2 | Concussion with moderate loss of consciousness |
| 850.3 | Concussion with prolonged loss of consciousness and return to pre-existing conscious level |
| 850.4 | Concussion with prolonged loss of consciousness, without return to pre-existing conscious level |
| 850.5 | Concussion with loss of consciousness of unspecified duration |
| 850.9 | Concussion, unspecified |
| 851.0 | Cortex (cerebral) contusion without mention of open intracranial wound |
| 851.00 | Cortex (cerebral) contusion without mention of open intracranial wound, unspecified state of consciousness |
| 851.01 | Cortex (cerebral) contusion without mention of open intracranial wound, with no loss of consciousness |
| 851.02 | Cortex (cerebral) contusion without mention of open intracranial wound, with brief [less than one hour] loss of consciousness |
| 851.03 | Cortex (cerebral) contusion without mention of open intracranial wound, with moderate [1-24 hours] loss of consciousness |
| 851.04 | Cortex (cerebral) contusion without mention of open intracranial wound, with prolonged [more than 24 hours] loss of consciousness and return to pre-existing conscious level |
| 851.05 | Cortex (cerebral) contusion without mention of open intracranial wound, with prolonged [more than 24 hours] loss of consciousness without return to pre-existing conscious level |
| 851.06 | Cortex (cerebral) contusion without mention of open intracranial wound, with loss of consciousness of unspecified duration |
| 851.09 | Cortex (cerebral) contusion without mention of open intracranial wound, with concussion, unspecified |
| 851.1 | Cortex (cerebral) contusion with open intracranial wound |
| 851.10 | Cortex (cerebral) contusion with open intracranial wound, unspecified state of consciousness |
| 851.11 | Cortex (cerebral) contusion with open intracranial wound, with no loss of consciousness |
| 851.12 | Cortex (cerebral) contusion with open intracranial wound, with brief [less than one hour] loss of consciousness |
| 851.13 | Cortex (cerebral) contusion with open intracranial wound, with moderate [1-24 hours] loss of consciousness |
| 851.14 | Cortex (cerebral) contusion with open intracranial wound, with prolonged [more than 24 hours] loss of consciousness and return to pre-existing conscious level |
| 851.15 | Cortex (cerebral) contusion with open intracranial wound, with prolonged [more than 24 hours] loss of consciousness without return to pre-existing conscious level |
| 851.16 | Cortex (cerebral) contusion with open intracranial wound, with loss of consciousness of unspecified duration |
| 851.19 | Cortex (cerebral) contusion with open intracranial wound, with concussion, unspecified |
| 851.2 | Cortex (cerebral) laceration without mention of open intracranial wound |
| 851.20 | Cortex (cerebral) laceration without mention of open intracranial wound, unspecified state of consciousness |
| 851.21 | Cortex (cerebral) laceration without mention of open intracranial wound, with no loss of consciousness |
| 851.22 | Cortex (cerebral) laceration without mention of open intracranial wound, with brief [less than one hour] loss of consciousness |
| 851.23 | Cortex (cerebral) laceration without mention of open intracranial wound, with moderate [1-24 hours] loss of consciousness |
| 851.24 | Cortex (cerebral) laceration without mention of open intracranial wound, with prolonged [more than 24 hours] loss of consciousness and return to pre-existing conscious level |
| 851.25 | Cortex (cerebral) laceration without mention of open intracranial wound, with prolonged [more than 24 hours] loss of consciousness without return to pre-existing conscious level |
| 851.26 | Cortex (cerebral) laceration without mention of open intracranial wound, with loss of consciousness of unspecified duration |
| 851.29 | Cortex (cerebral) laceration without mention of open intracranial wound, with concussion, unspecified |
| 851.3 | Cortex (cerebral) laceration with open intracranial wound |
| 851.30 | Cortex (cerebral) laceration with open intracranial wound, unspecified state of consciousness |
| 851.31 | Cortex (cerebral) laceration with open intracranial wound, with no loss of consciousness |
| 851.32 | Cortex (cerebral) laceration with open intracranial wound, with brief [less than one hour] loss of consciousness |
| 851.33 | Cortex (cerebral) laceration with open intracranial wound, with moderate [1-24 hours] loss of consciousness |
| 851.34 | Cortex (cerebral) laceration with open intracranial wound, with prolonged [more than 24 hours] loss of consciousness and return to pre-existing conscious level |
| 851.35 | Cortex (cerebral) laceration with open intracranial wound, with prolonged [more than 24 hours] loss of consciousness without return to pre-existing conscious level |
| 851.36 | Cortex (cerebral) laceration with open intracranial wound, with loss of consciousness of unspecified duration |
| 851.39 | Cortex (cerebral) laceration with open intracranial wound, with concussion, unspecified |
| 851.4 | Cerebellar or brain stem contusion without mention of open intracranial wound |
| 851.40 | Cerebellar or brain stem contusion without mention of open intracranial wound, unspecified state of consciousness |
| 851.41 | Cerebellar or brain stem contusion without mention of open intracranial wound, with no loss of consciousness |
| 851.42 | Cerebellar or brain stem contusion without mention of open intracranial wound, with brief [less than one hour] loss of consciousness |
| 851.43 | Cerebellar or brain stem contusion without mention of open intracranial wound, with moderate [1-24 hours] loss of consciousness |
| 851.44 | Cerebellar or brain stem contusion without mention of open intracranial wound, with prolonged [more than 24 hours] loss consciousness and return to pre-existing conscious level |
| 851.45 | Cerebellar or brain stem contusion without mention of open intracranial wound, with prolonged [more than 24 hours] loss of consciousness without return to pre-existing conscious level |
| 851.46 | Cerebellar or brain stem contusion without mention of open intracranial wound, with loss of consciousness of unspecified duration |
| 851.49 | Cerebellar or brain stem contusion without mention of open intracranial wound, with concussion, unspecified |
| 851.5 | Cerebellar or brain stem contusion with open intracranial wound |
| 851.50 | Cerebellar or brain stem contusion with open intracranial wound, unspecified state of consciousness |
| 851.51 | Cerebellar or brain stem contusion with open intracranial wound, with no loss of consciousness |
| 851.52 | Cerebellar or brain stem contusion with open intracranial wound, with brief [less than one hour] loss of consciousness |
| 851.53 | Cerebellar or brain stem contusion with open intracranial wound, with moderate [1-24 hours] loss of consciousness |
| 851.54 | Cerebellar or brain stem contusion with open intracranial wound, with prolonged [more than 24 hours] loss of consciousness and return to pre-existing conscious level |
| 851.55 | Cerebellar or brain stem contusion with open intracranial wound, with prolonged [more than 24 hours] loss of consciousness without return to pre-existing conscious level |
| 851.56 | Cerebellar or brain stem contusion with open intracranial wound, with loss of consciousness of unspecified duration |
| 851.59 | Cerebellar or brain stem contusion with open intracranial wound, with concussion, unspecified |
| 851.6 | Cerebellar or brain stem laceration without mention of open intracranial wound |
| 851.60 | Cerebellar or brain stem laceration without mention of open intracranial wound, unspecified state of consciousness |
| 851.61 | Cerebellar or brain stem laceration without mention of open intracranial wound, with no loss of consciousness |
| 851.62 | Cerebellar or brain stem laceration without mention of open intracranial wound, with brief [less than 1 hour] loss of consciousness |
| 851.63 | Cerebellar or brain stem laceration without mention of open intracranial wound, with moderate [1-24 hours] loss of consciousness |
| 851.64 | Cerebellar or brain stem laceration without mention of open intracranial wound, with prolonged [more than 24 hours] loss of consciousness and return to pre-existing conscious level |
| 851.65 | Cerebellar or brain stem laceration without mention of open intracranial wound, with prolonged [more than 24 hours] loss of consciousness without return to pre-existing conscious level |
| 851.66 | Cerebellar or brain stem laceration without mention of open intracranial wound, with loss of consciousness of unspecified duration |
| 851.69 | Cerebellar or brain stem laceration without mention of open intracranial wound, with concussion, unspecified |
| 851.7 | Cerebellar or brain stem laceration with open intracranial wound |
| 851.70 | Cerebellar or brain stem laceration with open intracranial wound, unspecified state of consciousness |
| 851.71 | Cerebellar or brain stem laceration with open intracranial wound, with no loss of consciousness |
| 851.72 | Cerebellar or brain stem laceration with open intracranial wound, with brief [less than one hour] loss of consciousness |
| 851.73 | Cerebellar or brain stem laceration with open intracranial wound, with moderate [1-24 hours] loss of consciousness |
| 851.74 | Cerebellar or brain stem laceration with open intracranial wound, with prolonged [more than 24 hours] loss of consciousness and return to pre-existing conscious level |
| 851.75 | Cerebellar or brain stem laceration with open intracranial wound, with prolonged [more than 24 hours] loss of consciousness without return to pre-existing conscious level |
| 851.76 | Cerebellar or brain stem laceration with open intracranial wound, with loss of consciousness of unspecified duration |
| 851.79 | Cerebellar or brain stem laceration with open intracranial wound, with concussion, unspecified |
| 851.8 | Other and unspecified cerebral laceration and contusion without mention of open intracranial wound |
| 851.80 | Other and unspecified cerebral laceration and contusion, without mention of open intracranial wound, unspecified state of consciousness |
| 851.81 | Other and unspecified cerebral laceration and contusion, without mention of open intracranial wound, with no loss of consciousness |
| 851.82 | Other and unspecified cerebral laceration and contusion, without mention of open intracranial wound, with brief [less than one hour] loss of consciousness |
| 851.83 | Other and unspecified cerebral laceration and contusion, without mention of open intracranial wound, with moderate [1-24 hours] loss of consciousness |
| 851.84 | Other and unspecified cerebral laceration and contusion, without mention of open intracranial wound, with prolonged [more than 24 hours] loss of consciousness and return to pre- existing conscious level |
| 851.85 | Other and unspecified cerebral laceration and contusion, without mention of open intracranial wound, with prolonged [more than 24 hours] loss of consciousness without return to pre-existing conscious level |
| 851.86 | Other and unspecified cerebral laceration and contusion, without mention of open intracranial wound, with loss of consciousness of unspecified duration |
| 851.89 | Other and unspecified cerebral laceration and contusion, without mention of open intracranial wound, with concussion, unspecified |
| 851.9 | Other and unspecified cerebral laceration and contusion with open intracranial wound |
| 851.90 | Other and unspecified cerebral laceration and contusion, with open intracranial wound, unspecified state of consciousness |
| 851.91 | Other and unspecified cerebral laceration and contusion, with open intracranial wound, with no loss of consciousness |
| 851.92 | Other and unspecified cerebral laceration and contusion, with open intracranial wound, with brief [less than one hour] loss of consciousness |
| 851.93 | Other and unspecified cerebral laceration and contusion, with open intracranial wound, with moderate [1-24 hours] loss of consciousness |
| 851.94 | Other and unspecified cerebral laceration and contusion, with open intracranial wound, with prolonged [more than 24 hours] loss of consciousness and return to pre-existing conscious level |
| 851.95 | Other and unspecified cerebral laceration and contusion, with open intracranial wound, with prolonged [more than 24 hours] loss of consciousness without return to pre-existing conscious level |
| 851.96 | Other and unspecified cerebral laceration and contusion, with open intracranial wound, with loss of consciousness of unspecified duration |
| 851.99 | Other and unspecified cerebral laceration and contusion, with open intracranial wound, with concussion, unspecified |
| 852.0 | Subarachnoid hemorrhage following injury without mention of open intracranial wound |
| 852.00 | Subarachnoid hemorrhage following injury without mention of open intracranial wound, unspecified state of consciousness |
| 852.01 | Subarachnoid hemorrhage following injury without mention of open intracranial wound, with no loss of consciousness |
| 852.02 | Subarachnoid hemorrhage following injury without mention of open intracranial wound, with brief [less than one hour] loss of consciousness |
| 852.03 | Subarachnoid hemorrhage following injury without mention of open intracranial wound, with moderate [1-24 hours] loss of consciousness |
| 852.04 | Subarachnoid hemorrhage following injury without mention of open intracranial wound, with prolonged [more than 24 hours] loss of consciousness and return to pre-existing conscious level |
| 852.05 | Subarachnoid hemorrhage following injury without mention of open intracranial wound, with prolonged [more than 24 hours] loss of consciousness without return to pre-existing conscious level |
| 852.06 | Subarachnoid hemorrhage following injury without mention of open intracranial wound, with loss of consciousness of unspecified duration |
| 852.09 | Subarachnoid hemorrhage following injury without mention of open intracranial wound, with concussion, unspecified |
| 852.1 | Subarachnoid hemorrhage following injury with open intracranial wound |
| 852.10 | Subarachnoid hemorrhage following injury with open intracranial wound, unspecified state of consciousness |
| 852.11 | Subarachnoid hemorrhage following injury with open intracranial wound, with no loss of consciousness |
| 852.12 | Subarachnoid hemorrhage following injury with open intracranial wound, with brief [less than one hour] loss of consciousness |
| 852.13 | Subarachnoid hemorrhage following injury with open intracranial wound, with moderate [1-24 hours] loss of consciousness |
| 852.14 | Subarachnoid hemorrhage following injury with open intracranial wound, with prolonged [more than 24 hours) loss of consciousness and return to pre-existing conscious level |
| 852.15 | Subarachnoid hemorrhage following injury with open intracranial wound, with prolonged [more than 24 hours] loss of consciousness without return to pre-existing conscious level |
| 852.16 | Subarachnoid hemorrhage following injury with open intracranial wound, with loss of consciousness of unspecified duration |
| 852.19 | Subarachnoid hemorrhage following injury with open intracranial wound, with concussion, unspecified |
| 852.2 | Subdural hemorrhage following injury without mention of open intracranial wound |
| 852.20 | Subdural hemorrhage following injury without mention of open intracranial wound, unspecified state of consciousness |
| 852.21 | Subdural hemorrhage following injury without mention of open intracranial wound, with no loss of consciousness |
| 852.22 | Subdural hemorrhage following injury without mention of open intracranial wound, with brief [less than one hour] loss of consciousness |
| 852.23 | Subdural hemorrhage following injury without mention of open intracranial wound, with moderate [1-24 hours] loss of consciousness |
| 852.24 | Subdural hemorrhage following injury without mention of open intracranial wound, with prolonged [more than 24 hours] loss of consciousness and return to pre-existing conscious level |
| 852.25 | Subdural hemorrhage following injury without mention of open intracranial wound, with prolonged [more than 24 hours] loss of consciousness without return to pre-existing conscious level |
| 852.26 | Subdural hemorrhage following injury without mention of open intracranial wound, with loss of consciousness of unspecified duration |
| 852.29 | Subdural hemorrhage following injury without mention of open intracranial wound, with concussion, unspecified |
| 852.3 | Subdural hemorrhage following injury with open intracranial wound |
| 852.30 | Subdural hemorrhage following injury with open intracranial wound, unspecified state of consciousness |
| 852.31 | Subdural hemorrhage following injury with open intracranial wound, with no loss of consciousness |
| 852.32 | Subdural hemorrhage following injury with open intracranial wound, with brief [less than one hour] loss of consciousness |
| 852.33 | Subdural hemorrhage following injury with open intracranial wound, with moderate [1-24 hours] loss of consciousness |
| 852.34 | Subdural hemorrhage following injury with open intracranial wound, with prolonged [more than 24 hours] loss of consciousness and return to pre-existing conscious level |
| 852.35 | Subdural hemorrhage following injury with open intracranial wound, with prolonged [more than 24 hours] loss of consciousness without return to pre-existing conscious level |
| 852.36 | Subdural hemorrhage following injury with open intracranial wound, with loss of consciousness of unspecified duration |
| 852.39 | Subdural hemorrhage following injury with open intracranial wound, with concussion, unspecified |
| 852.4 | Extradural hemorrhage following injury without mention of open intracranial wound |
| 852.40 | Extradural hemorrhage following injury without mention of open intracranial wound, unspecified state of consciousness |
| 852.41 | Extradural hemorrhage following injury without mention of open intracranial wound, with no loss of consciousness |
| 852.42 | Extradural hemorrhage following injury without mention of open intracranial wound, with brief [less than 1 hour] loss of consciousness |
| 852.43 | Extradural hemorrhage following injury without mention of open intracranial wound, with moderate [1-24 hours] loss of consciousness |
| 852.44 | Extradural hemorrhage following injury without mention of open intracranial wound, with prolonged [more than 24 hours] loss of consciousness and return to pre-existing conscious level |
| 852.45 | Extradural hemorrhage following injury without mention of open intracranial wound, with prolonged [more than 24 hours] loss of consciousness without return to pre-existing conscious level |
| 852.46 | Extradural hemorrhage following injury without mention of open intracranial wound, with loss of consciousness of unspecified duration |
| 852.49 | Extradural hemorrhage following injury without mention of open intracranial wound, with concussion, unspecified |
| 852.5 | Extradural hemorrhage following injury with open intracranial wound |
| 852.50 | Extradural hemorrhage following injury with open intracranial wound, unspecified state of consciousness |
| 852.51 | Extradural hemorrhage following injury with open intracranial wound, with no loss of consciousness |
| 852.52 | Extradural hemorrhage following injury with open intracranial wound, with brief [less than one hour] loss of consciousness |
| 852.53 | Extradural hemorrhage following injury with open intracranial wound, with moderate [1-24 hours] loss of consciousness |
| 852.54 | Extradural hemorrhage following injury with open intracranial wound, with prolonged [more than 24 hours] loss of consciousness and return to pre-existing conscious level |
| 852.55 | Extradural hemorrhage following injury with open intracranial wound, with prolonged [more than 24 hours] loss of consciousness without return to pre-existing conscious level |
| 852.56 | Extradural hemorrhage following injury with open intracranial wound, with loss of consciousness of unspecified duration |
| 852.59 | Extradural hemorrhage following injury with open intracranial wound, with concussion, unspecified |
| 853.0 | Other and unspecified intracranial hemorrhage following injury without mention of open intracranial wound |
| 853.00 | Other and unspecified intracranial hemorrhage following injury without mention of open intracranial wound, unspecified state of consciousness |
| 853.01 | Other and unspecified intracranial hemorrhage following injury without mention of open intracranial wound, with no loss of consciousness |
| 853.02 | Other and unspecified intracranial hemorrhage following injury without mention of open intracranial wound, with brief [less than one hour] loss of consciousness |
| 853.03 | Other and unspecified intracranial hemorrhage following injury without mention of open intracranial wound, with moderate [1-24 hours] loss of consciousness |
| 853.04 | Other and unspecified intracranial hemorrhage following injury without mention of open intracranial wound, with prolonged [more than 24 hours] loss of consciousness and return to pre- existing conscious level |
| 853.05 | Other and unspecified intracranial hemorrhage following injury without mention of open intracranial wound, with prolonged [more than 24 hours] loss of consciousness without return to pre-existing conscious level |
| 853.06 | Other and unspecified intracranial hemorrhage following injury without mention of open intracranial wound, with loss of consciousness of unspecified duration |
| 853.09 | Other and unspecified intracranial hemorrhage following injury without mention of open intracranial wound, with concussion, unspecified |
| 853.1 | Other and unspecified intracranial hemorrhage following injury with open intracranial wound |
| 853.10 | Other and unspecified intracranial hemorrhage following injury with open intracranial wound, unspecified state of consciousness |
| 853.11 | Other and unspecified intracranial hemorrhage following injury with open intracranial wound, with no loss of consciousness |
| 853.12 | Other and unspecified intracranial hemorrhage following injury with open intracranial wound, with brief [less than one hour] loss of consciousness |
| 853.13 | Other and unspecified intracranial hemorrhage following injury with open intracranial wound, with moderate [1-24 hours] loss of consciousness |
| 853.14 | Other and unspecified intracranial hemorrhage following injury with open intracranial wound, with prolonged [more than 24 hours] loss of consciousness and return to pre-existing conscious level |
| 853.15 | Other and unspecified intracranial hemorrhage following injury with open intracranial wound, with prolonged [more than 24 hours] loss of consciousness without return to pre-existing conscious level |
| 853.16 | Other and unspecified intracranial hemorrhage following injury with open intracranial wound, with loss of consciousness of unspecified duration |
| 853.19 | Other and unspecified intracranial hemorrhage following injury with open intracranial wound, with concussion, unspecified |
| 854.0 | Intracranial injury of other and unspecified nature without mention of open intracranial wound |
| 854.00 | Intracranial injury of other and unspecified nature without mention of open intracranial wound, unspecified state of consciousness |
| 854.01 | Intracranial injury of other and unspecified nature without mention of open intracranial wound, with no loss of consciousness |
| 854.02 | Intracranial injury of other and unspecified nature without mention of open intracranial wound, with brief [less than one hour] loss of consciousness |
| 854.03 | Intracranial injury of other and unspecified nature without mention of open intracranial wound, with moderate [1-24 hours] loss of consciousness |
| 854.04 | Intracranial injury of other and unspecified nature without mention of open intracranial wound, with prolonged [more than 24 hours] loss of consciousness and return to pre-existing conscious level |
| 854.05 | Intracranial injury of other and unspecified nature without mention of open intracranial wound, with prolonged [more than 24 hours] loss of consciousness without return to pre-existing conscious level |
| 854.06 | Intracranial injury of other and unspecified nature without mention of open intracranial wound, with loss of consciousness of unspecified duration |
| 854.09 | Intracranial injury of other and unspecified nature without mention of open intracranial wound, with concussion, unspecified |
| 854.1 | Intracranial injury of other and unspecified nature with open intracranial wound |
| 854.10 | Intracranial injury of other and unspecified nature with open intracranial wound, unspecified state of consciousness |
| 854.11 | Intracranial injury of other and unspecified nature with open intracranial wound, with no loss of consciousness |
| 854.12 | Intracranial injury of other and unspecified nature with open intracranial wound, with brief [less than one hour] loss of consciousness |
| 854.13 | Intracranial injury of other and unspecified nature with open intracranial wound, with moderate [1-24 hours] loss of consciousness |
| 854.14 | Intracranial injury of other and unspecified nature with open intracranial wound, with prolonged [more than 24 hours] loss of consciousness and return to pre-existing conscious level |
| 854.15 | Intracranial injury of other and unspecified nature with open intracranial wound, with prolonged [more than 24 hours] loss of consciousness without return to pre-existing conscious level |
| 854.16 | Intracranial injury of other and unspecified nature with open intracranial wound, with loss of consciousness of unspecified duration |
| 854.19 | Intracranial injury of other and unspecified nature with open intracranial wound, with concussion, unspecified |
