# Supplementary material for: Diagnostic conversion to bipolar disorder among adolescents and young adults with major depressive disorder: a nationwide longitudinal study
Source: Eur Child Adolesc Psychiatry. 2024 Mar 29;33(10):3625–35. doi: 10.1007/s00787-024-02401-1 (PMC11564236; doi:10.1007/s00787-024-02401-1)
Supplement: Supplementary file 2 — Supplementary file2 (DOCX 34 KB) [file 787_2024_2401_MOESM2_ESM.docx]

**Supplemental Table 2. The rationales for the study variables**

| **Variables** | **Rationale** | **Reference** |
| --- | --- | --- |
| Age | A previous national study in Korea (E. Y. Kim et al., 2020) indicated that the conversion rate was highest among individuals aged 18–29 years, gradually decreasing with age until reaching 60–69 years.  Our study also revealed that a younger age at the diagnosis of major depressive disorder (MDD) posed a risk factor for bipolar disorder (BD) conversion, aligning with findings from this study. | Kim, E. Y., Kim, N. W., Kim, M. J., Yang, B. R., Rhee, S. J., Park, C. H. K., Lee, H. J., Kim, S. H., & Ahn, Y. M. 2020. Rate of diagnostic conversion to bipolar disorder in adults with unipolar depression and psychopharmacological treatment in the republic of Korea: A nationwide register-based study. *Journal of Affective Disorders*, 273(240-246). |
| Sex | The findings are inconsistent for the associations between sex and BD conversion. While large national studies in England (James et al., 2015) and Korea (Kim et al., 2020b) reported that sex was a risk factor for diagnostic conversion to BD, most studies, including ours, did not show significance (Gilman et al., 2012; Oliveira et al., 2021). | James, A., Wotton, C. J., Duffy, A., Hoang, U. & Goldacre, M. 2015. Conversion from depression to bipolar disorder in a cohort of young people in England, 1999-2011: A national record linkage study. *Journal of Affective Disorders*, 185(123-8).  Kim, H., Kim, Y., Baek, J. H., Fava, M., Mischoulon, D., Nierenberg, A. A., Choi, K. W., Na, E. J., Shin, M. H. & Jeon, H. J. 2020b. Predictive factors of diagnostic conversion from major depressive disorder to bipolar disorder in young adults ages 19-34: A nationwide population study in South Korea. *Journal of Affective Disorders*, 265(52-58).  Gilman, S. E., Dupuy, J. M., & Perlis, R. H. 2012. Risks for the transition from major depressive disorder to bipolar disorder in the National Epidemiologic Survey on Alcohol and Related Conditions. *Journal of Clinical Psychiatry*, 73(6), 829-836.  Oliveira, J. P., Jansen, K., Cardoso, T. A., Mondin, T. C., Souza, L. D. M., Silva, R. A. D., & Pedrotti Moreira, F. 2021. Predictors of conversion from major depressive disorder to bipolar disorder. *Psychiatry Research*, 297, 113740. |
| Antidepressant resistance | One of the most important risk factors associated with diagnostic conversion to BD is the level of antidepressant resistance, indicating inadequate response despite at least two trials of antidepressants at adequate doses and durations (Rush et al., 2006). Treatment resistance not only escalates the medical burden and individual and social costs but also stands as a significant predictor of bipolar disorder conversion (Li et al., 2012). Alternatively, these MDD patients exhibiting antidepressant resistance may represent latent BD cases or a subsyndromal manifestation, warranting avoidance of antidepressant monotherapy and consideration of mood stabilizers or certain atypical antipsychotics (Rihmer et al., 2016). | Rush, A. J., Trivedi, M. H., Wisniewski, S. R., Nierenberg, A. A., Stewart, J. W., Warden, D., Niederehe, G., Thase, M. E., Lavori, P. W., Lebowitz, B. D., McGrath, P. J., Rosenbaum, J. F., Sackeim, H. A., Kupfer, D. J., Luther, J., & Fava, M. 2006. Acute and longer-term outcomes in depressed outpatients requiring one or several treatment steps: a STAR*D report. *The American journal of psychiatry*, 163(11), 1905–1917.  Li, C. T., Bai, Y. M., Huang, Y. L., Chen, Y. S., Chen, T. J., Cheng, J. Y. & Su, T. P. 2012. Association between antidepressant resistance in unipolar depression and subsequent bipolar disorder: cohort study. *The British Journal of Psychiatry*, 200(1), pp 45-51.  Rihmer, Z., Gonda, X., Rihmer, A., & Dome, P. 2016. [Antidepressant-resistant depression and the bipolar spectrum -- diagnostic and therapeutic considerations]. *Psychiatria Hungarica*, 31(2), 157-168. |
| Physical comorbidities | | |
| Epilepsy | A meta-analysis in 2013 suggested that the prevalence of depression among individuals with epilepsy was as high as 23% in certain studies (Fiest et al., 2013). Both epilepsy and cerebrovascular disease exhibit bidirectional relationships with MDD, implying shared pathogenic mechanisms between these two physical comorbidities and MDD (Kanner, 2006; Gothe et al., 2012). Another possibility relates to the use of antiepileptic drugs, commonly employed as mood stabilizers in the treatment of BD (Bialer, 2012), thereby reducing the risk of manic episodes (Viktorin et al., 2014). | Fiest, K. M., Dykeman, J., Patten, S. B., Wiebe, S., Kaplan, G. G., Maxwell, C. J., Bulloch, A. G., & Jette, N. 2013. Depression in epilepsy: a systematic review and meta-analysis. *Neurology*, 80(6), 590-599.  Kanner, A. M. 2006. Depression and epilepsy: a new perspective on two closely related disorders. *Epilepsy Currents*, 6(5), pp 141-6.  Gothe, F., Enache, D., Wahlund, L. O., Winblad, B., Crisby, M., Lokk, J. & Aarsland, D. 2012. Cerebrovascular diseases and depression: epidemiology, mechanisms and treatment. *Panminerva Medica*, 54(3), pp 161-70.  Bialer, M. 2012. Why are antiepileptic drugs used for nonepileptic conditions? *Epilepsia*, 53 Suppl 7(26-33).  Viktorin, A., Lichtenstein, P., Thase, M. E., Larsson, H., Lundholm, C., Magnusson, P. K. & Landen, M. 2014. The risk of switch to mania in patients with bipolar disorder during treatment with an antidepressant alone and in combination with a mood stabilizer. *American Journal of Psychiatry*, 171(10), pp 1067-73 |
| Autoimmune disease | A nationwide prospective cohort study in Denmark uncovered that autoimmune diseases and infections serve as risk factors for a subsequent diagnosis of mood disorders (Benros et al., 2013). | Benros, M. E., Waltoft, B. L., Nordentoft, M., Østergaard, S. D., Eaton, W. W., Krogh, J., & Mortensen, P. B. 2013. Autoimmune Diseases and Severe Infections as Risk Factors for Mood Disorders: A Nationwide Study. *JAMA Psychiatry*, 70(8):812–820. |
| Atopic disease | A nationwide longitudinal study conducted in Taiwan discovered an association between atopic diseases during adolescence and the subsequent risk of developing mood disorders (Wei et al., 2016). | Wei, H. T., Lan, W. H., Hsu, J. W., Huang, K. L., Su, T. P., Li, C. T., Lin, W. C., Chen, T. J., Bai, Y. M., & Chen, M. H. 2016. Risk of developing major depression and bipolar disorder among adolescents with atopic diseases: A nationwide longitudinal study in Taiwan. *Journal of affective disorders*, 203, 221–226. |
| Thyroid disease | Depression and cognitive dysfunction are among the most prevalent psychiatric symptoms associated with hypothyroidism (Bauer et al., 2008). However, a systematic review and meta-analysis in 2021 not revealing a clear, significant link between hypothyroidism and depression (Bode et al., 2021). We aim to further clarify the relationship between thyroid disorders and bipolar disorder in adolescents and young adults. | Bauer, M., Goetz, T., Glenn, T., & Whybrow, P. C. 2008. The thyroid-brain interaction in thyroid disorders and mood disorders. Journal of neuroendocrinology, 20(10), 1101–1114.  Bode H, Ivens B, Bschor T, Schwarzer G, Henssler J, & Baethge C. 2021. Association of Hypothyroidism and Clinical Depression: A Systematic Review and Meta-analysis. *JAMA Psychiatry*, 78(12):1375–1383. |
| Cerebrovascular disease | Both epilepsy and cerebrovascular disease have bidirectional relationships with MDD, suggesting common pathogenic mechanisms between these two physical comorbidities and MDD (Kanner, 2006; Gothe et al, 2012). | Kanner, A. M. 2006. Depression and epilepsy: a new perspective on two closely related disorders. *Epilepsy Currents*, 6(5), pp 141-6.  Gothe, F., Enache, D., Wahlund, L. O., Winblad, B., Crisby, M., Lokk, J. & Aarsland, D. 2012. Cerebrovascular diseases and depression: epidemiology, mechanisms and treatment. *Panminerva Medica*, 54(3), pp 161-70. |
| Traumatic brain injury (TBI) | The neuropathological changes induced by TBI may lead to the deactivation of the dorsolateral prefrontal cortex and the activation of ventral limbic and paralimbic structures, including the amygdala. These changes play a significant role in the complex pathophysiology of MDD (Jorge et al., 2004). | Jorge, R. E., Robinson, R. G., Moser, D., Tateno, A., Crespo-Facorro, B., & Arndt, S. 2004. Major depression following traumatic brain injury. *Archives Of General Psychiatry*, 61(1), 42-50. |
| Obesity | Previous studies have suggested a bidirectional relationship between obesity and mood disorders in adults (Simon et al., 2006; Mansur et al., 2015). The National Comorbidity Survey Replication reported that individuals with obesity had 1.47-fold greater odds of lifetime BD (Simon et al., 2006; Restivo et al., 2016). TCF7L2 has been identified as a risk allele associated with BD susceptibility and higher body mass index (BMI) (Winham et al., 2014), which might explain this relationship. Furthermore, obesity has been proposed to lead to antidepressant resistance in individuals with MDD (Toups & Trivedi, 2011) and exacerbate the severity of MDD, consequently increasing the risk of BD (Musliner & Ostergaard, 2018). | Simon, G. E., Von Korff, M., Saunders, K., Miglioretti, D. L., Crane, P. K., van Belle, G. & Kessler, R. C. 2006. Association between obesity and psychiatric disorders in the US adult population. *Archives Of General Psychiatry,* 63(7), pp 824-30.  Mansur, R. B., Brietzke, E. & McIntyre, R. S. 2015. Is there a "metabolic-  mood syndrome"? A review of the relationship between obesity and mood  disorders. *Neuroscience & Biobehavioral Reviews,* 52(89-104).  Restivo, M. R., McKinnon, M. C., Frey, B. N., Hall, G. B. & Taylor, V. H. 2016. Effect of obesity on cognition in adults with and without a mood disorder: study design and methods. *BMJ Open,* 6(2), pp e009347.  Winham, S. J., Cuellar-Barboza, A. B., Oliveros, A., McElroy, S. L., Crow, S., Colby, C., Choi, D. S., Chauhan, M., Frye, M. & Biernacka, J. M. 2014. Genome-wide association study of bipolar disorder accounting for effect of body mass index identifies a new risk allele in TCF7L2. *Molecular Psychiatry,* 19(9), pp 1010-6.  Toups, M. S., & Trivedi, M. H. 2011. Role of metabolic dysfunction in treatment resistance of major depressive disorder. *Neuropsychiatry*, 1(5), 441–455.  Musliner, K. L. & Ostergaard, S. D. 2018. Patterns and predictors of  Conversion to bipolar disorder in 91 587 individuals diagnosed with  unipolar depression. *Acta Psychiatrica Scandinavica,* 137(5), pp 422-432. |
| Psychiatric comorbidities | | |
| Attention deficit/hyperactivity disorder (ADHD) | Episodes of MDD occurring alongside ADHD increase the likelihood of conversion from MDD to BD (Chen et al., 2015), possibly due to sub-threshold symptoms of BD and basic-level emotional dysregulation, as proposed by Biederman et al. (Biederman et al., 2014). A review article from 2006 offered several explanations for the co-occurrence of both disorders: ADHD might serve as the prodromal stage of early-onset BD, treatment for ADHD such as psychostimulants might trigger the onset of BD, or both disorders might potentially share a common pathogenesis (Singh et al., 2006). | Chen, M. H., Chen, Y. S., Hsu, J. W., Huang, K. L., Li, C. T., Lin, W. C., Chang, W. H., Chen, T. J., Pan, T. L., Su, T. P., & Bai, Y. M. 2015. Comorbidity of ADHD and subsequent bipolar disorder among adolescents and young adults with major depression: a nationwide longitudinal study. *Bipolar Disorder*, 17(3), 315-322.  Biederman, J., Wozniak, J., Tarko, L., Serra, G., Hernandez, M., McDermott, K., Woodsworth, K. Y., Uchida, M., & Faraone, S. V. 2014. Re-examining the risk for switch from unipolar to bipolar major depressive disorder in youth with ADHD: a long term prospective longitudinal controlled study. *Journal of Affective Disorders*, 152-154, 347-351.  Singh, M. K., DelBello, M. P., Kowatch, R. A., & Strakowski, S. M. 2006. Co-occurrence of bipolar and attention-deficit hyperactivity disorders in children. *Bipolar Disorders*, 8(6), 710-720. |
| Alcohol use disorder (AUD) | The epidemiological data from the US indicate high rates of comorbidities in bipolar disorder (BD), with lifetime prevalence rates reported up to 90%. Among these comorbidities, 62.3% are associated with AUD, comprising 39.1% for DSM-IV alcohol abuse and 23.2% for alcohol dependency (Merikangas et al., 2007).  Also, individuals diagnosed with bipolar disorder face a greater risk of developing alcohol use disorder compared to the general population (Helzer et al., 1988). | Merikangas, K. R., Akiskal, H. S., Angst, J., Greenberg, P. E., Hirschfeld, R. M., Petukhova, M., & Kessler, R. C. 2007. Lifetime and 12-month prevalence of bipolar spectrum disorder in the National Comorbidity Survey replication. *Archives of general psychiatry*, 64(5), 543–552.  Helzer, J. E., & Pryzbeck, T. R. 1988. The co-occurrence of alcoholism with other psychiatric disorders in the general population and its impact on treatment. *Journal of studies on alcohol*, 49(3), 219–224. |
| Substance use disorder (SUD) | The literature has suggested a familial association between BD and SUD (Lin et al., 2006), implying a potential genetic relationship. Additionally, another study proposed that intermittent stressors could act as precipitating factors for both BD and SUD, as they displayed cross-sensitization (Post & Kalivas, 2013). Affective instability, mood dysregulation, and impaired impulse control are prominent factors in the development of BD (Mesbah et al., 2021). | Lin, P. I., McInnis, M. G., Potash, J. B., Willour, V., MacKinnon, D. F., DePaulo, J. R., & Zandi, P. P. 2006. Clinical correlates and familial aggregation of age at onset in bipolar disorder. *American Journal of Psychiatry*, 163(2), 240-246.  Post, R. M., & Kalivas, P. 2013. Bipolar disorder and substance misuse: pathological and therapeutic implications of their comorbidity and cross-sensitization. *The British Journal of Psychiatry*, 202(3), 172-176.  Mesbah, R., de Bles, N., Rius-Ottenheim, N., van der Does, A. J. W., Penninx, B. W. J. H., van Hemert, A. M., de Leeuw, M., Giltay, E. J., & Koenders, M. 2021. Anger and cluster B personality traits and the conversion from unipolar depression to bipolar disorder. *Depression and Anxiety*, *38*(6), 671-681. |
| Smoking | Several authors have put forward the argument that tobacco smoking leads to inferior treatment outcomes in bipolar disorder (Corvin et al., 2001). However, contrasting research findings exist (Kreinin et al., 2012), where certain researchers have not discovered a clear relationship between the two. We aim to further clarify the relationship between smoking and bipolar disorder in adolescents and young adults. | Corvin, A., O'Mahony, E., O'Regan, M., Comerford, C., O'Connell, R., Craddock, N., & Gill, M. 2001. Cigarette smoking and psychotic symptoms in bipolar affective disorder. *The British journal of psychiatry: the journal of mental science*, 179, 35–38.  Kreinin, A., Novitski, D., Rabinowitz, D., Weizman, A., & Grinshpoon, A. (2012). Association between tobacco smoking and bipolar affective disorder: clinical, epidemiological, cross-sectional, retrospective study in outpatients. *Comprehensive psychiatry*, 53(3), 269–274. |
| Posttraumatic stress disorder (PTSD) | Individuals with bipolar disorder face an increased risk of experiencing traumatic events, possibly due to disruptive and disorganized behavior during manic episodes. The prevalence of PTSD among individuals with bipolar disorder varied between 4% and 40%, and women diagnosed with bipolar I disorder show a higher likelihood of experiencing PTSD (Cerimele et al., 2017). | Cerimele, J. M., Bauer, A. M., Fortney, J. C., & Bauer, M. S. 2017. Patients With Co-Occurring Bipolar Disorder and Posttraumatic Stress Disorder: A Rapid Review of the Literature. *The Journal of clinical psychiatry*, 78(5), e506–e514. |
| Cluster A personality disorder (PD) | We want to compare the relationship between different types of personality disorders and bipolar disorder in adolescents and young adults. |  |
| Cluster B PD | Our findings align with the aforementioned studies referencing SUD and cluster B personality disorder (PD) (Kim et al., 2020; Oliveira et al., 2021), suggesting that affective instability, mood dysregulation, and impaired impulse control in behavior play a prominent role in the development of bipolar disorder (Mesbah et al., 2021). | Kim, E. Y., Kim, N. W., Kim, M. J., Yang, B. R., Rhee, S. J., Park, C. H. K., Lee, H. J., Kim, S. H. & Ahn, Y. M. 2020a. Rate of diagnostic conversion to bipolar disorder in adults with unipolar depression and psychopharmacological treatment in the republic of Korea: A nationwide register-based study. *Journal of Affective Disorders*, 273(240-246).  Oliveira, J. P., Jansen, K., Cardoso, T. A., Mondin, T. C., Souza, L. D. M., Silva, R. A. D., & Pedrotti Moreira, F. (2021). Predictors of conversion from major depressive disorder to bipolar disorder. *Psychiatry Research*, 297, 113740.  Mesbah, R., de Bles, N., Rius-Ottenheim, N., van der Does, A. J. W., Penninx, B. W. J. H., van Hemert, A. M., de Leeuw, M., Giltay, E. J., & Koenders, M. 2021. Anger and cluster B personality traits and the conversion from unipolar depression to bipolar disorder. *Depression and Anxiety*, *38*(6), 671-681. |
| Cluster C PD | Our study revealed an association between cluster C PDs and BD conversion, a correlation that has not been previously explored. Past studies have indicated that obsessive-compulsive PD is frequently linked to BD (Rossi et al., 2001; Casas-Barquero et al., 2007). Given that this disorder falls within cluster C PDs, it could potentially explain our findings. | Rossi, A., Marinangeli, M. G., Butti, G., Scinto, A., Di Cicco, L., Kalyvoka, A., & Petruzzi, C. 2001. Personality disorders in bipolar and depressive disorders. *Journal of Affective Disorders*, *65*(1), 3-8.  Casas-Barquero, N., Garcia-Lopez, O., Fernandez-Arguelles, P., & Camacho-Larana, M. 2007. Clinical variables and implications of the personality on the outcome of bipolar illness: a pilot study. *Neuropsychiatric Disease and Treatment*, *3*(2), 269-275. |
| Family history | | |
| Schizophrenia | Among family histories of mental disorders at baseline, the most robust associations were identified for schizophrenia and BD, aligning with previous research that highlighted a significant relationship between the degree of kinship and the heritability of bipolar disorder (Escamilla & Zavala, 2008). This suggests a shared familial vulnerability between schizophrenia and BD, as indicated by Aukes et al. in 2012 (Aukes et al., 2012). | Escamilla, M. A., & Zavala, J. M. 2008. Genetics of bipolar disorder. *Dialogues in Clinical Neuroscience*, *10*(2), 141-152.  Aukes, M. F., Laan, W., Termorshuizen, F., Buizer-Voskamp, J. E., Hennekam, E. A. M., Smeets, H. M., Ophoff, R. A., Boks, M. P. M., & Kahn, R. S. 2012. Familial clustering of schizophrenia, bipolar disorder, and major depressive disorder. *Genetics in Medicine*, *14*(3), 338-341. |
| Bipolar disorder (BD) | Among family histories of mental disorders at baseline, the most robust associations were identified for schizophrenia and BD, aligning with previous research that highlighted a significant relationship between the degree of kinship and the heritability of bipolar disorder (Escamilla & Zavala, 2008). This suggests a shared familial vulnerability between schizophrenia and BD, as indicated by Aukes et al. in 2012 (Aukes et al., 2012). | Escamilla, M. A., & Zavala, J. M. 2008. Genetics of bipolar disorder. *Dialogues in Clinical Neuroscience*, *10*(2), 141-152.  Aukes, M. F., Laan, W., Termorshuizen, F., Buizer-Voskamp, J. E., Hennekam, E. A. M., Smeets, H. M., Ophoff, R. A., Boks, M. P. M., & Kahn, R. S. 2012. Familial clustering of schizophrenia, bipolar disorder, and major depressive disorder. *Genetics in Medicine*, *14*(3), 338-341. |
| Major depressive disorder (MDD) | Compared to schizophrenia and BD, MDD is more susceptible to environmental factors (Aukes et al., 2012). This explains the outcome in our study, indicating that a family history of schizophrenia and BD exhibits a stronger relationship with the conversion to BD. | Aukes, M. F., Laan, W., Termorshuizen, F., Buizer-Voskamp, J. E., Hennekam, E. A. M., Smeets, H. M., Ophoff, R. A., Boks, M. P. M., & Kahn, R. S. 2012. Familial clustering of schizophrenia, bipolar disorder, and major depressive disorder. *Genetics in Medicine*, *14*(3), 338-341. |
| Attention deficit/hyperactivity disorder (ADHD) | As episodes of MDD occurring alongside ADHD increase the likelihood of conversion from MDD to BD (Chen et al., 2015), we would like to investigate the contribution of a family history of ADHD to conversion to BD in adolescents and young adults. | Chen, M. H., Chen, Y. S., Hsu, J. W., Huang, K. L., Li, C. T., Lin, W. C., Chang, W. H., Chen, T. J., Pan, T. L., Su, T. P., & Bai, Y. M. 2015. Comorbidity of ADHD and subsequent bipolar disorder among adolescents and young adults with major depression: a nationwide longitudinal study. Bipolar Disorder, 17(3), 315-322. |
| Residential location | Monthly income levels (≤15,840 New Taiwanese dollars (NTD), 15,841-25,000 NTD, and ≥25,000 NTD) and residential location (levels 1-5, from most to least urbanized) were considered proxies for healthcare availability in Taiwan (Liu et al., 2006).  Regarding the level of urbanization, our study's results are mixed, possibly limited by our national territorial area, making it challenging to distinctly differentiate the most urbanized areas from the most rural ones due to minimal discrimination. | Liu, C. Y., Hung, Y. T., Chuang, Y. L., Chen, Y. J., Weng, W. S. & Liu, J. S. 2006. Incorporating development stratification of Taiwan townships into sampling design of large scale health interview survey. *Journal of Health Management (Chin),* 4(1-22). |
| Monthly income | Monthly income levels (≤15,840 New Taiwanese dollars (NTD), 15,841-25,000 NTD, and ≥25,000 NTD) and residential location (levels 1-5, from most to least urbanized) were considered proxies for healthcare availability in Taiwan (Liu et al., 2006).  In a prospective, longitudinal, nationally representative survey conducted by Jitender Sareen et al., involving a total of 34,653 participants followed up for 3 years, a conclusion was drawn that a decline in income correlates with an increased risk of developing mental disorders (Sareen et al., 2011), which aligns with our findings. | Liu, C. Y., Hung, Y. T., Chuang, Y. L., Chen, Y. J., Weng, W. S. & Liu, J. S. 2006. Incorporating development stratification of Taiwan townships into sampling design of large scale health interview survey. *Journal of Health Management (Chin),* 4(1-22).  Sareen, J., Afifi, T. O., McMillan, K. A., & Asmundson, G. J. G. 2011. Relationship Between Household Income and Mental Disorders: Findings From a Population-Based Longitudinal Study. *Archives of General Psychiatry*, *68*(4), 419-427. |
| Frequency of clinical visits for mental health per year | Frequency of clinical visits for mental health issues is considered to be associated with the diagnostic conversion to Bipolar Disorder (BD), an intriguing phenomenon noted by Ágnes Lublóy et al. termed “bipolar diagnostic delay” (Lublóy et al., 2020). Frequent visits to mental health clinics might indicate instability and a lack of illness insight. Moreover, we included this variable to account for detection bias. However, the association between these two variables (frequency of clinical visits and diagnostic conversion to BD) may not be substantial, as there was only a small increase in the hazard ratio. Besides, we do not believe that controlling for the frequency of clinical visits would make the difference between converters and non-converters vanish. Therefore, the probability of detection bias might be trivial. | Lublóy, Á., Keresztúri, J. L., Németh, A., & Mihalicza, P. 2020. Exploring factors of diagnostic delay for patients with bipolar disorder: a population-based cohort study. *BMC Psychiatry*, *20*(1), 75. |
